# Molecular Dynamics Insights into the Biodegradation of Synthetic Polymers by *Moniliophthora roreri* Cutinases

**DOI:** 10.1021/acs.jcim.5c03051

**Published:** 2026-03-24

**Authors:** Maycon Vinicius Damasceno de Oliveira, Gabriel Calandrini, Carlos Gabriel da Silva de Souza, Clauber H. S. da Costa, Munir S. Skaf, Jerônimo Lameira

**Affiliations:** † Laboratório de Planejamento e Desenvolvimento de Fármacos, Instituto de Ciências Exatas e Naturais, 37871 Universidade Federal do Pará, Belém, Pará 66075-110, Brasil; ‡ Programa de Pós-Graduação em Ecologia Aquática e Pesca (PPGEAP), Universidade Federal do Pará, Belém, Pará 66075-110, Brasil; § Institute of Chemistry and Center for Computing in Engineering & Sciences, 28132 University of Campinas–UNICAMP. Campinas, São Paulo 13084-862, Brazil

## Abstract

The natural degradation of synthetic polymers is an extremely slow process, often taking hundreds to thousands of years. The fungus *Moniliophthora roreri* produces cutinase enzymes, such as *Mr*Cut1, *Mr*Cut2, and *Mr*Cut3, which are capable of depolymerizing synthetic polymers. Although experimental studies have demonstrated that the *Mr*Cut1 enzyme can degrade polyethylene succinate (PES), polycaprolactone (PCL), and polyethylene terephthalate (PET), its three-dimensional structure and the atomistic details underlying the binding process with these polymers remain elusive. Here, we present the first atomistic simulations of *Mr*Cut1 in complex with PES, PCL, and PET oligomers, providing structural insights into substrate recognition, binding modes, and key interactions driving polymer degradation. Additionally, we evaluated the structural differences among *Mr*Cut1, *Mr*Cut2, and *Mr*Cut3 to assess how these variations affect their affinity for forming enzyme–polymer complexes. Through Free Energy Landscape (FEL) and active site volume analyses, we demonstrate the structural similarity between *Mr*Cut1 and *Mr*Cut3, as well as their respective volumes for polymer binding at the active site. Our results indicate that the binding affinities of PES, PCL, and PET for *Mr*Cut1 are −9.17 ± 0.49, −9.00 ± 0.56, and −8.40 ± 0.49 kcal/mol, respectively. In contrast, these ligands exhibit lower affinities for *Mr*Cut3, with values of −7.99 ± 0.43, −7.95 ± 0.60, and −7.30 ± 0.42 kcal/mol, respectively. Furthermore, our findings suggest that the absence of the catalytic triad in *Mr*Cut2, together with the lack of a well-formed active site cavity, may result in its inefficient catalytic activity. Finally, we demonstrate, for the first time, the potential of *Mr*Cut3 as a biocatalyst, similar to *Mr*Cut1, and explore its applicability in the biodegradation of certain synthetic polymers.

## Graphical abstract

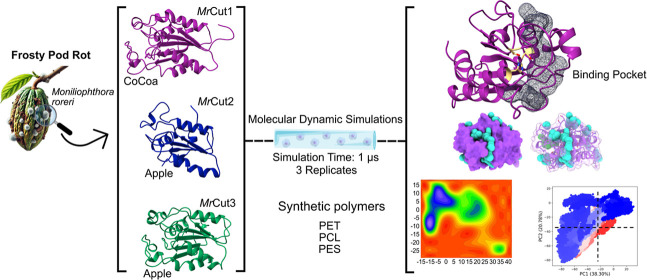

## Introduction

Plastic pollution is a major global environmental issue that impacts both land and marine ecosystems, posing serious threats to human health and biodiversity.^1,2^ The widespread use of plastics and inadequate waste management practices have led to the pervasive presence of plastic debris in the environment. Due to its low cost, polyethylene terephthalate (PET) is extensively used in the production of bottles and other materials, making it the most abundantly produced plastic material, which consequently contributes significantly to environmental degradation.^1–5^

The biodegradation of synthetic polymers is a very slow process and can take hundreds to thousands of years.^6^ However, enzymes such as cutinases and PETases, for example, accelerate the degradation of these compounds via hydrolysis, depolymerization, or oxidation. These enzymes can be produced naturally by microorganisms, including fungi and bacteria, or synthesized via biotechnological methods.^6–10^

Cutinase is a hydrolytic enzyme belonging to the hydrolase family, capable of degrading cutin, a natural polyester present in the plant cuticle.^11,12^ Recent studies have reported its ability to act on synthetic polymers, like PET, due to its open structure and exposed active site. Its structure and catalytic mechanism are similar to those of PETase, an enzyme that evolved more recently to specifically degrade PET. Both enzymes share the same catalytic triad (Ser-His-Asp) and are capable of cleaving ester bonds in polymeric substrates.^13–15^ However, PETase exhibits higher efficiency in the amorphous regions of PET, where increased chain mobility enhances enzymatic access and bond cleavage. In these regions, the enzyme progresses more readily along the polymer, accelerating overall hydrolysis. Conversely, rigid or crystalline domains restrict accessibility and markedly reduce catalytic performance. Additionally, PETase activity shows a clear positive correlation with the mobile amorphous fraction of the substrat.^16,17^

Structural modifications in PETases have proven effective in enhancing their ability to degrade PET, especially in its amorphous form. These modifications, often guided by molecular simulations, reveal that flexibility in specific regions of the enzyme facilitates the recognition and binding of the polymer at the active site. This more efficient enzyme–substrate interaction improves conformational stability and increases catalytic performance. Thus, the combination of structural dynamics and rational engineering strengthens the development of more efficient enzymes for applications in plastic biodegradation.^15,18,19^

Among microorganisms capable of producing enzymes that degrade synthetic polymers, the fungus *Moniliophthora roreri* (family *Marasmiaceae*) has been identified as a source of the cutinase enzyme (*Mr*Cut1). Additionally, two other cutinase genes have been found in its genome (*Mr*Cut2 and *Mr*Cut3). *M. roreri* can degrade polymers such as polyethylene succinate (PES), polycaprolactone (PCL), and polyethylene terephthalate (PET). This fungus is also known to cause Frosty Pod Disease (FPD), a condition that affects cocoa plantations (*Theobroma cacao*) by contaminating the fruit, leading to significant economic losses in the cocoa industry, especially in Brazil.^20–24^ During the 20th century, Brazil was the largest cocoa producer in the world. However, due to the increasing number of diseases such as witches’ broom on plantations, Brazil lost this title as the world’s largest producer. As of 2020, Brazil remains among the world’s top 10 cocoa bean producers.^25–28^

In addition to *M. roreri*, other microorganisms, such as the fungus *Aspergillus nidulans* and the bacterium *Thermobifida fusca*, have also been reported as producers of two or more cutinases.^29–31^ *M. roreri* produces three cutinases: *Mr*Cut1, *Mr*Cut2, and *Mr*Cut3.^32^ The *Mr*Cut1 gene have been overexpressed in *Escherichia coli* and *Mr*Cut1 has been evaluated for its ability to degrade synthetic polyesters such as PET, PES and PCL.^32^

In this study, we present a molecular level analysis of the interactions and complex formation between synthetic polymers (PET, PES, and PCL) and the cutinases *Mr*Cut1, *Mr*Cut2, and *Mr*Cut3. Since the three-dimensional structures of these proteins have not yet been elucidated by experimental techniques, we modeled their structures and performed molecular dynamics simulations, Free Energy Landscape (FEL) analysis, and binding free energy calculations. We compare our results with a recent experimental study for *Mr*Cut1 ^32^ and elucidate the structural determinants of polymer binding affinity. Additionally, we assess the potential of *Mr*Cut3, alongside the previously characterized *Mr*Cut1, as biocatalysts for the degradation of synthetic polymers.

## Materials and Methods

### Protocol for Obtaining and Structural Preparation of Cutinases *Mr*Cut1, *Mr*Cut2 and *Mr*Cut3 from *M. roreri*

The fungus *M. roreri* produces three types of cutinases under different conditions: *Mr*Cut1 (Cocoa cuticle), *Mr*Cut2 (Apple cuticle) and *Mr*Cut3 (Apple cuticle).^32^ The sequences of the cutinases were obtained from GenBank, with the following codes: *Mr*Cut1 (ESK97883.1), *Mr*Cut2 (ESK95146), *Mr*Cut3 (ESK92300.1) (Table S1). The amino acid sequences were aligned with cutinase enzymes from different organisms to identify conserved regions and common motifs of the enzyme. The BLAST^33^ was used to select the organisms and retrieve the corresponding sequences, while the MEGA^34^ software was employed to perform the multiple sequence alignment. The three-dimensional structures of the proteins were obtained through Swiss-model^35^ (Figure S1).

### Protocol for Preparation of Complexes and Simulation by Molecular Dynamics

The protonation states of the ionizable amino acids of the cutinases *Mr*Cut1, *Mr*Cut2 and *Mr*Cut3 were determined through the free online server H^++^ ^15,18,36^ that uses the p*K*_a_ calculation assuming a specific pH value, in this case 8.0 ^32^. The ff14SB force field was used to describe the amino acids of the proteins. The complexes were solvated with TIP3P^37^ water molecules (explicit solvation mode), in an octahedral water box of 12 Å. We added Na^+^ counterions to maintain the electroneutrality of the systems only for *Mr*Cut2 and *Mr*Cut3. We used four steps to perform the structural energy minimization. In all steps, AMBER initially applies steepest descent and then switches to conjugate gradient according to the ncyc and maxcyc parameters. In the first step, only the counterions and water molecules were allowed to move (ibelly = 1), using a total of 8000 steps (4000 steepest descent +4000 conjugate gradient) with a drms convergence of 0.001 kcal·mol^–1^·Å^–1^. In the second step, only the protein hydrogen atoms were minimized for 5000 steps (2000 + 3000). In the third step, we minimized the protein hydrogens together with the water molecules, again for 8000 steps using the same drms criterion. Finally, in the fourth step, we performed a full minimization of the entire system (ibelly = 0) without restraints, using 10,000 steps (5000 + 5000). This gradual protocol allowed the system to relax smoothly before the heating and equilibration phases. We started the process of heating the systems from 0 to 313.15 K using 200 ps of MD at constant volume with a restraint weight of 5.0 kcal·mol^–1^ Å^2^ on the positions of the atoms (complex). The next step was to equilibrate the systems with 500 ps of unrestricted MD at a constant pressure of 1 bar. In this step, the temperature of the systems was maintained at 313.15 K using a Langevin thermostat with a collision frequency of 2 ps^–1^ and assuming an isotropic constant pressure of 1 bar using a Berendsen barostat. A cutoff of 10 Å was applied to all nonbonded interactions, and the Particle Mesh Ewald (PME) method was employed to treat long-range electrostatics. Subsequently, 1 μs production runs were performed for each system in the *NPT* ensemble. For each model system, three independent 1 μs runs were performed, totaling 9 μs of extensive MD simulations. All MD simulations were performed using *pmemd* program available in Amber22.^38^ Fingerprint interaction analyses were conducted with the ProfLIF program^40^ using the entire simulation time. To refine the results, all frames from each simulation were included in the analysis.

We first performed PCA and FEL analyses using the apo trajectories. From the minimum energy region, we selected the structure in which the active site cavity was clearly defined, and this model was used as the starting point for the molecular docking stage. The *Mr*Cut1 and *Mr*Cut3 complexes with the synthetic polymers PCL, PES, and PET were generated (Table 1) using Molegro Virtual Docker, following the same protocol described in our previous studies.^15,18^ The three polymers were parametrized using the CHARMM-GUI^39–41^ web server with the CHARMM36 ^42^ force field, as in our earlier work.^15,18^ For each protein–polymer complex, five independent MD replicates of 300 ns were performed, totaling 1.5 μs of simulation for each polymer system.

**Table 1:**
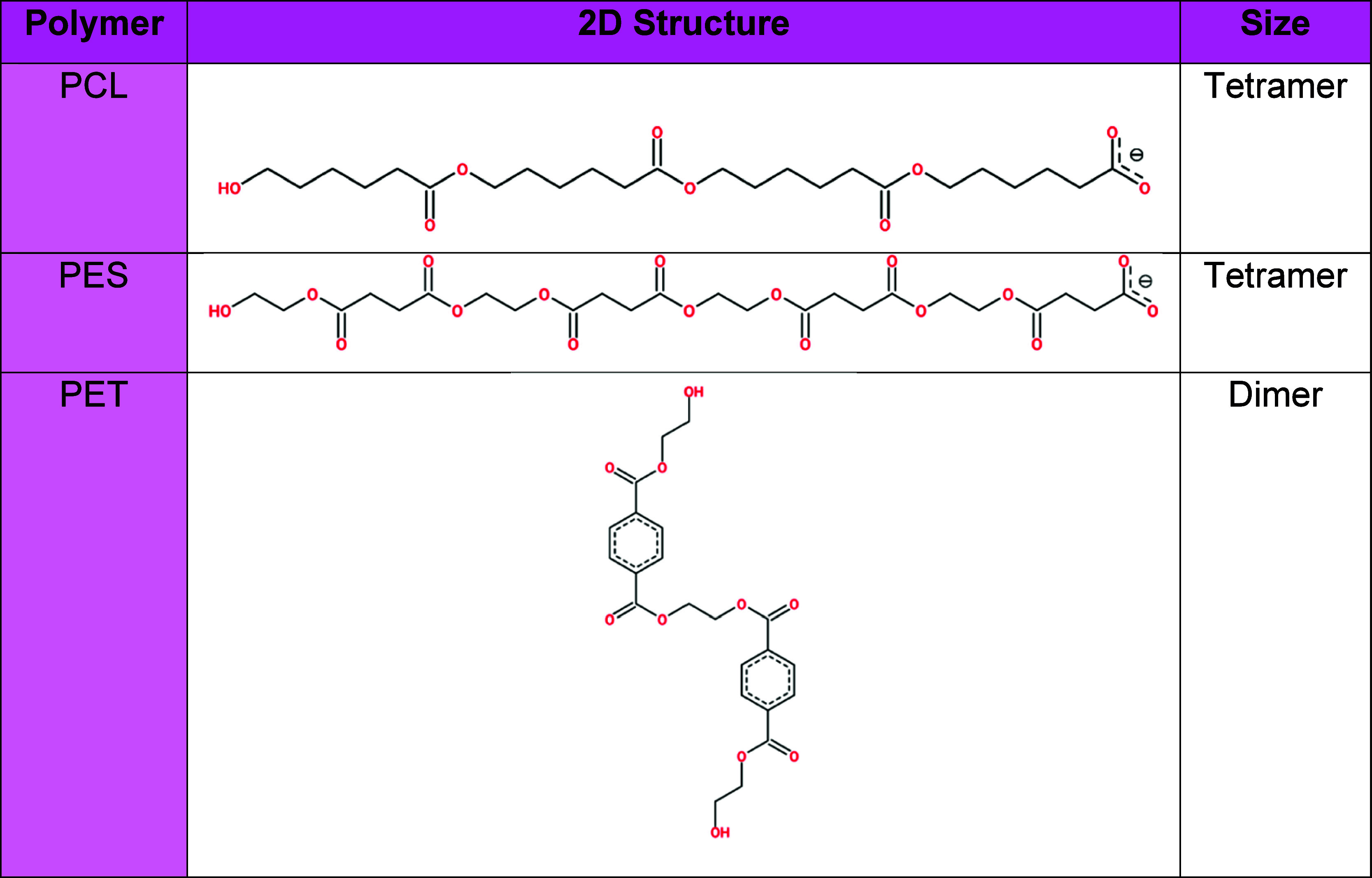
Oligomers Representing Synthetic Polymers: Polyethylene Succinate (PES), Polycaprolactone (PCL), and Polyethylene Terephthalate (PET)

## Results and Discussion

### The Three-Dimensional Model of the *Mr*Cut1, *Mr*Cut2 and *Mr*Cut3 Proteins

The three-dimensional protein models were constructed using the Swiss-Model server using the cutinase from the fungus *Trichoderma reesei* (PDB ID: 4PSC) as a structural template,^43^ which shares a sequence identity of 43.96% with *Mr*Cut1, 46.49% with *Mr*Cut2, and 44.63% with *Mr*Cut3, as determined by local alignment using the BLAST server.^33^ Phylogenetic analysis between *T. reesei* and *M. roreri* further supports this choice, revealing a clade with a bootstrap value of 95, indicative of strong statistical support for a recent common ancestor within the group of filamentous fungi.^15^ The obtained model exhibits high stereochemical quality, with over 94% of residues located in favored regions (Table S3). Furthermore, the Swiss-Model server automatically trims poorly modeled regions at the N- and C- terminal ends, further improving the structural reliability of the predicted models as observed in crystallographic structures of cutinases from other organisms deposited in the PDB.^43–46^ The models constructed with Swiss-Model were selected for the subsequent Molecular Dynamics simulation protocols.

### Analysis of the Structural Similarity of the Enzymes *Mr*Cut1, *Mr*Cut2 and *Mr*Cut3 and the Similarity with the Cutinase of *T. reesei*

An initial point of comparison among the enzymes is their total number of amino acid residues, which are 194, 174, and 195 for *Mr*Cut1, *Mr*Cut2, and *Mr*Cut3, respectively. Notably, the cutinase expressed by the fungus *T. reesei* (used as the template) is one of the few fungal cutinases that contains an additional disulfide bond, possessing three disulfide bridges in total.^54^ In contrast, *Mr*Cut1 and *Mr*Cut3 have two disulfide bridges each, while *Mr*Cut2 contains only a single disulfide bridge (Figure 1).

**Figure 1:**
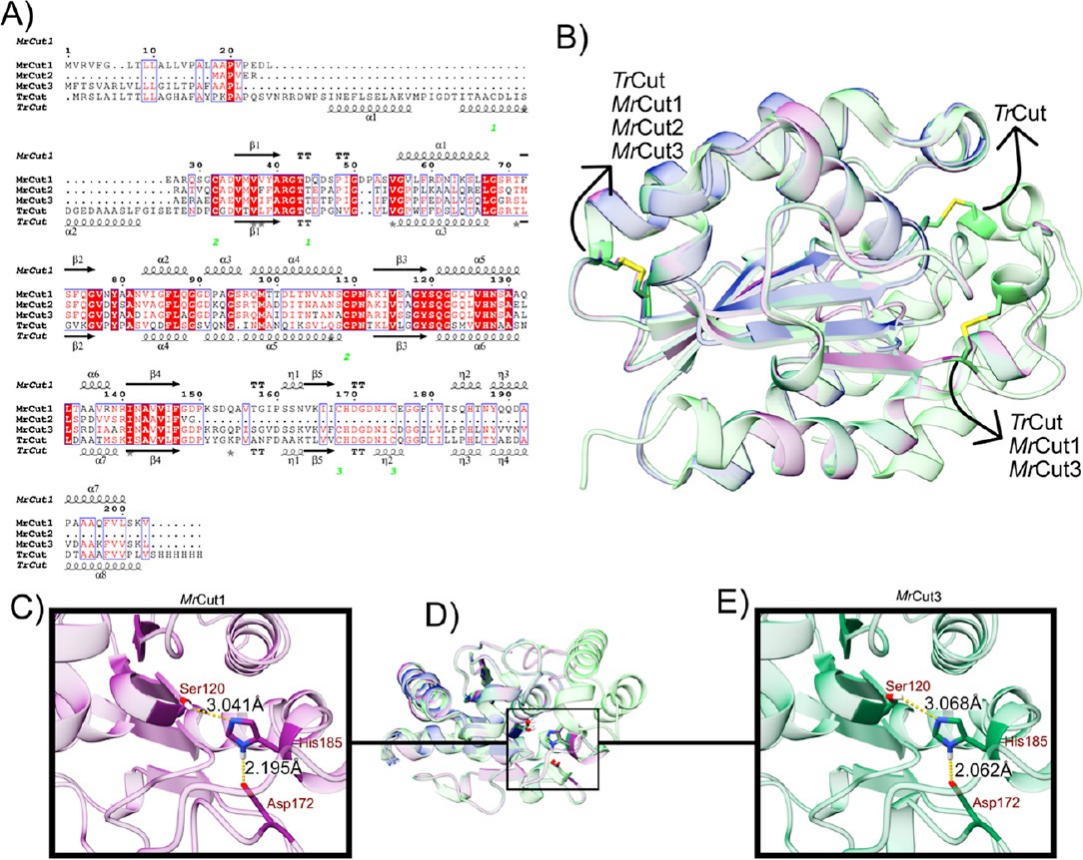
Similarity analysis between the enzymes *Mr*Cut1, *Mr*Cut2 and *Mr*Cut3 in comparison with the enzyme *Tr*Cut (template) through (A) amino acid sequence alignment using Clustal Omega, (B) enzyme overlap, (C–E) overlap of the catalytic triad Ser-His-Asp.

The number of disulfide bridges in bacterial cutinases is generally lower than in fungal cutinases, as observed in organisms such as *T. fusca* (PDB ID: 5ZOA), *Thermobifida cellulosilytica* (PDB ID: 5LUI),^47^ *Thermobifida alba* (PDB ID: 3VIS),^48^ *Saccharomonosphora flava* (PDB ID: 7QJP),^49^ *Saccharomonosphora viridis* (PDB ID: 4WFI)^46^ and *Halopseudomonas bauzanensis* (PDB ID: 8AIT).^50^ While fungi such as *T. reesei* (PDB ID: 4PSC),^43^ *Aspergillus oryzae* (PDB ID: 3GBS)^51^ and *Malbranchea cinnamomea* (PDB ID: 5X88)^52^ have three disulfide bridges in their structure. Furthermore, the additional disulfide bridge in the Cutinase of the fungus *A. oryzae* favors thermostability in the biodegradation process of the PCL polymer.^53^

The first disulfide bridge of *Tr*Cut, Cys55–Cys91, involves a region near the N-terminus of the protein. In addition, *Tr*Cut consists of 250 amino acids, with the primary difference compared to the *M. roreri* cutinase located in the enzyme’s N-terminal region (Figure 1), where the additional disulfide bridge is located. The enzymes *Tr*Cut, *Mr*Cut1, and *Mr*Cut3 share the same two disulfide bridges in conserved regions, while the single disulfide bridge of *Mr*Cut2 is also located in the corresponding conserved region. These observations demonstrate that *Mr*Cut1, *Mr*Cut2, *Mr*Cut3, and *Tr*Cut maintain conservation of disulfide bridges in these conserved regions (Figure 1B). The enzymes *Mr*Cut1 and *Mr*Cut3 bear two disulfide bridges in their structures, similar to the cutinase from the fungus *Fusarium solani*. In this organism the disulfide bridges play a fundamental role in its structural stability.^45,54,55^ Moreover, the cutinase enzyme from *T. fusca* has greater thermostability due to the disulfide bond in its structure.^56^

The catalytic triad Ser-His-Asp is present in the same region for *Tr*Cut (Ser164-His229-Asp216), *Mr*Cut1 and *Mr*Cut3 (Ser120-His185-Asp172). This cleaving ester bonds in the natural polymer cutin and in synthetic polymers such as PET. The nucleophilic attack is mediated by the catalytic serine, similarly to what is observed in PETase. Compared to *Mr*Cut1 and *Mr*Cut3, the *Mr*Cut2 enzyme has a smaller structure, which affects its overall conformation and catalytic function, as it lacks a complete catalytic triad and contains only the catalytic serine. This may be related to the fact that the genes encoded in the apple cuticle for *Mr*Cut2 and *Mr*Cut3 are 500 and 600 bp long, respectively,^32^ where these sizes are not consistent with the expected DNA size, which can be justified by the presence of introns in these genes.^32^

### MD Simulations

During the simulations, the *Mr*Cut1 enzyme remained stable during replicate 1, replicate 2 and replicate 3 (Figure 2A) with average RMSD values being 2.15 ± 0.36 Å, 1.76 ± 0.22 Å and 2.33 ± 0.53 Å, respectively. In replicate 3, from 960 ns onward the RMSD increases to a scale of 4.0 due to the movement of the protein loops. The catalytic region of *Mr*Cut1 remained stable during the simulation, similar to what was observed for *Mr*Cut3.

**Figure 2:**
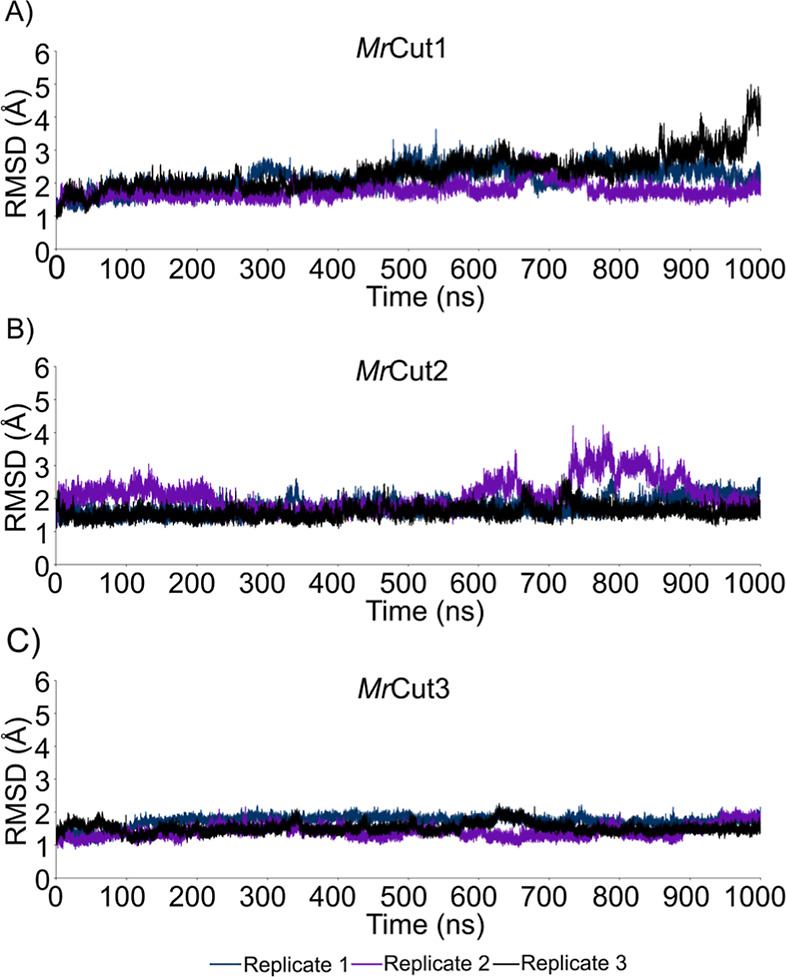
RMSD plot (atoms CA, C, O, N), in Å, of the ligand free (A) *Mr*Cut1, (B) *Mr*Cut2 and (C) *Mr*Cut3 enzymes during the entire MD simulation time of 1000 ns (1us) for each of the replicates.

For *Mr*Cut2, the system remained stable across all replicates (Figure 2B); however, replicate 2 exhibited an RMSD exceeding 2.5 Å between 600 ns and approximately 900 ns of simulation, which can be attributed to conformational fluctuations of loops near the enzyme’s active site. *Mr*Cut2 lacks the complete Ser–His–Asp catalytic triad, containing only the catalytic serine, which results in an active site entirely distinct from those of other fungal and bacterial cutinases. Despite this variation, the simulations of *Mr*Cut2 remained stable, with average RMSD values of 1.74 ± 0.24 Å, 2.10 ± 0.47 Å, and 1.58 ± 0.18 Å for replicates 1, 2, and 3, respectively. Only in replicate 2 the average value slightly exceeded 2 Å but remained within the stability threshold of 3 Å.

Among the enzymes analyzed, *Mr*Cut3 exhibited the highest stability across all three replicates throughout the entire simulation period (Figure 2C). Consequently, the movement of the protein in the three independent replicates was extremely similar, maintaining the monomer structure preserved. In addition, the active site of the protein remained stable during the simulation. The average RMSD values for each replicate demonstrate that the enzyme *Mr*Cut3 is the most stable among the observed systems with 1.72 ± 0.16 Å, 1.40 ± 0.19 Å and 1.51 ± 0.15 Å, respectively.

Therefore, the RMSD plots show that *Mr*Cut1 remains stable in the 1.5–3.0 Å range, reaching a plateau after 150 ns, with only one replicate (Replicate 3) displaying a noticeable increase near 900 ns. For *Mr*Cut2, two replicates stay consistently stable around 2 Å throughout the simulation, while one replicate (Replicate 2) shows fluctuations between 500 and 800 ns but eventually returns to the stable baseline toward the end. *Mr*Cut3 is the most consistent variant, with all replicates stabilized between 1.5 and 2.5 Å across the entire trajectory and no relevant deviations. Overall, the three systems exhibit stable structural.

Here, we also used RMSF analysis to map regions of amino acid fluctuation to characterize protein movement during MD simulations. For the *Mr*Cut1 enzyme (Figure 3A,D), three loop regions were identified. In region 1, the main residues are nonpolar (Pro and Ile); in region 2, they are polar; and in region 3, both polar (Gly and Glu) and nonpolar (Phe and Ile) residues are present. The structures of *Mr*Cut1 and *Mr*Cut3 have two disulfide bridges in contrast to *Mr*Cut2, which has a smaller conformation and a single disulfide bridge, referring to I-disulfide bridge of *Mr*Cut1 and *Mr*Cut3. I-disulfide bridge appears to be responsible for linking the amino (N-terminal) region of the protein to its central region (Figure 1B). The II-disulfide bond is associated with the catalytic loop of the enzyme, which maintains the conformational integrity of the catalytic triad. In this region the histidine of the catalytic triad is involved, as observed in Cutinase from other organisms such as *T. reesei*,^43^ *F. solani*,^44^ *Fusarium oxysporum*,^45^ *Kineococcus radiotolerans*.^57^ This loop is entirely related to the possibility of the substrate entering the catalytic site, exposing the region of the site to capture the substrate.

**Figure 3:**
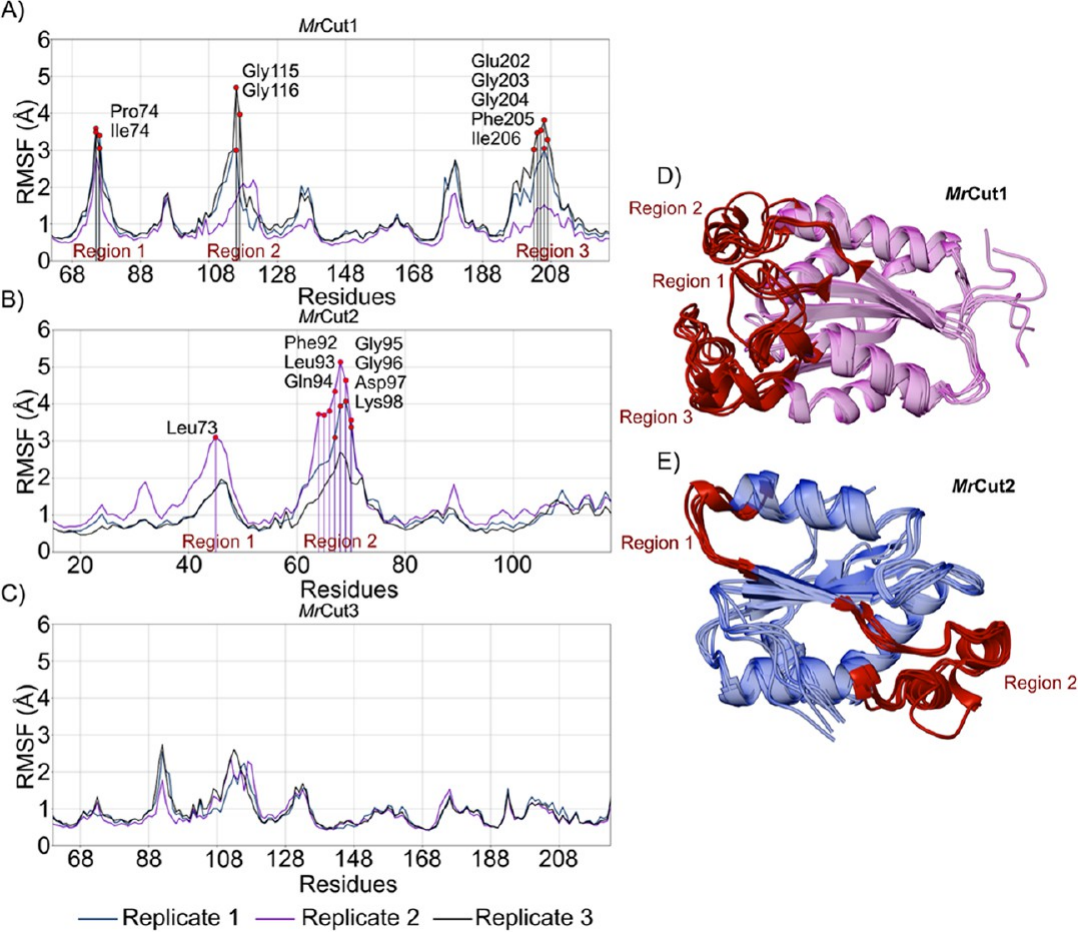
RMSF analysis for (A) *Mr*Cut1, (B) *Mr*Cut2 and (C) *Mr*Cut3 highlighting the regions of fluctuation in the enzyme (D) *Mr*Cut1 and (E) *Mr*Cut2.

In the *Mr*Cut1 simulations, Region 1 is located near the I-disulfide bridge. In the disulfide bridge analysis, the highest values were observed for replicates 1 and 3, which also showed greater fluctuations in this region. In Region 2, the I-disulfide bridge may also influence the mobility of the loop, as there are only 18 amino acids preceding this region. Lastly, region 3 refers to the catalytic loop where the II-Disulfide Bridge and histidine of the catalytic triad stand out. Region 3 exhibits pronounced fluctuations in the loop residues in Replicas 1 and 3 (Figure 3). Consistent with the observations from the analysis of the second disulfide bond, the increase in the average RMSD is directly associated with the mobility of the enzyme’s catalytic loop. This loop plays a key role in modulating the accessibility of the catalytic site, as its movement facilitates substrate entrance by promoting an “opening” of the active site. These findings provide valuable insights for studies investigating the use of cutinase as a biocatalyst in the biodegradation of polymers such as PET, PCL, and related materials.

For the *Mr*Cut2 systems, the highlighted regions (Figure 3B) are involved by the I-disulfide bond. Data from the analysis of the I-disulfide bond are correlated with the fluctuation of residues. The highest values of the average RMSD are for Replica 1 and 2 and the RMSF graph presents the largest fluctuations for the same replicas.

*Mr*Cut3 did not exhibit any regions with fluctuations equal to or greater than 3 Å. Correlation of the disulfide bond and RMSF analyses indicates that the catalytic loop of *Mr*Cut3 undergoes minimal conformational fluctuations, suggesting that its active site does not adopt the open conformation observed in *Mr*Cut1. The catalytic loop does not move to allow substrate access to the enzyme’s active site. This provides one of the first explanations for why only the *Mr*Cut1 enzyme, despite its similarity to *Mr*Cut3, has been reported among the three studied enzymes to biodegrade synthetic polymers.^32^

### Free Energy Landscape Analysis

The PCA projections onto the first two principal components (PC1 and PC2) reveal pronounced differences in conformational heterogeneity among the three simulated systems (Figure S2). For *Mr*Cut1, PC1 and PC2 explain 38.3% and 20.8% of the total variance, respectively, resulting in a broad distribution across the PCA space. The presence of multiple high-density subregions separated by low-probability areas suggests the sampling of distinct conformational substates. This interpretation is reinforced by the 1D histograms of PC1 and PC2, which display clearly multimodal patterns indicative of transitions between structurally differentiated conformations along the trajectory. Such enhanced heterogeneity is consistent with the FEL results (Figure 4), which exhibit several well-defined energy minima, supporting the existence of multiple energetically accessible conformational states.

**Figure 4:**
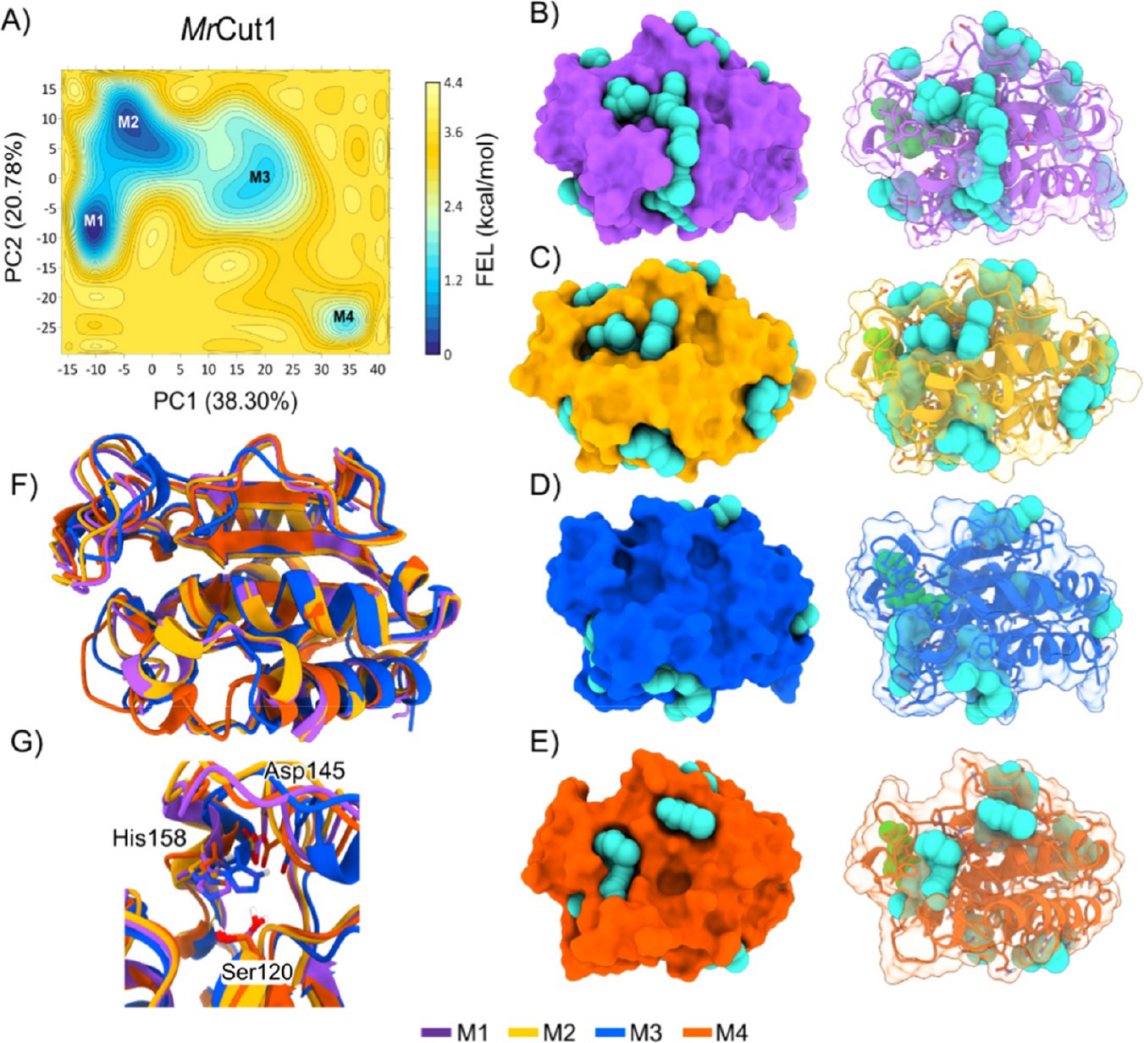
(A) Free energy landscape (FEL) result for *Mr*Cut1, with the energy surface in kcal/mol mapped as a function of the principal components PC1 and PC2. The representative structures of each minimum are shown, highlighting the pocket volumes (in cyan spheres) for each structure. (B) Structure corresponding to minimum M1. (C) Structure corresponding to minimum M2. (D) Structure corresponding to minimum M3. (E) Structure corresponding to minimum M4. (F) Comparison of the structures of minima M1, M2, M3 and M4. (G) Alignment of the regions of the catalytic triad.

In contrast, *Mr*Cut2 displays a markedly more compact conformational ensemble. PC1 accounts for 55.3% of the total variance, while PC2 contributes only 9.8%, indicating that conformational fluctuations are predominantly governed by a single collective mode. The PCA projection is concentrated around a single major minimum with limited dispersion along PC2. The corresponding 1D histograms further support this behavior, exhibiting narrow and mostly unimodal distributions for both components, suggestive of fewer substates and reduced transitions throughout the simulation. This trend aligns with the FEL analysis, which reveals fewer minima located closer to one another in energetic terms.

*Mr*Cut3 exhibits the most restricted conformational sampling among the three systems. Although PC1 and PC2 account for 39.1% and 11.1% of the variance, respectively, the actual dispersion in the PCA projection is minimal. The associated histograms show narrow distributions, reflecting local fluctuations around a single dominant state with no evidence of significant conformational rearrangements. This behavior is consistent with its FEL profile, which contains only shallow and energetically similar minima, reinforcing the more rigid structural nature of this system. The remaining PCs are shown in Figure S3, where PC1 vs PC2, PC2 vs PC3, and PC3 vs PC1 projections are presented for *Mr*Cut1 (Figure S3A), *Mr*Cut2 (Figure S3B), and *Mr*Cut3 (Figure S3C).

Free energy landscape (FEL) analysis identified four energy minima for *Mr*Cut1, with Minimum M1 exhibiting the lowest free energy, indicating higher stability and predominance of this conformation during the molecular dynamics (MD) simulation. Minima M2, M3, and M4 correspond to higher-energy states. The representative structure of M1 (Figure 4B) reveals a substantially larger cavity in the active site region, suggesting an enhanced capacity to accommodate substrates such as PET. This cavity has the largest volume among the identified states, measuring 1068.0 Å^3^, while the volumes of the other minima are listed in Table S4 (Figure 4C–E). The alternative minima exhibit less stable conformations with smaller cavities, which expand over time and eventually reach the maximum volume observed in M1.

The FEL analysis indicates that M1 is the most probable conformation for substrate binding, highlighting that molecular dynamics simulations allow structural changes that expand the active site, making it more accessible to the substrate. This increase in cavity volume demonstrates that the protein undergoes a series of conformational changes, involving states M2, M3, and M4, until reaching the most stable state, M1, while preserving the catalytic triad, as shown in Figure 4G.

MD simulations revealed that *Mr*Cut2 does not form a well-defined cavity in the region of the potential active site where Ser120 is located (Figure 5G). The four energy minima (M5, M6, M7, and M8) shown in the FEL (Figure 5A) exhibit conformational stability states that do not provide a sufficiently wide-open cavity to directly accommodate the PET polymer (see Figure 5B–E). Since PET is composed of dimeric monomers forming an elongated structure, the lack of a clear cavity in the lowest energy minima hinders substrate binding. Even with the presence of Ser120 in the active site, as shown in Figure 5G, the absence of the catalytic triad in *Mr*Cut2, and the lack of a well-formed cavity in the active site region, leads to the absence of efficient catalytic activity in this enzyme.

**Figure 5:**
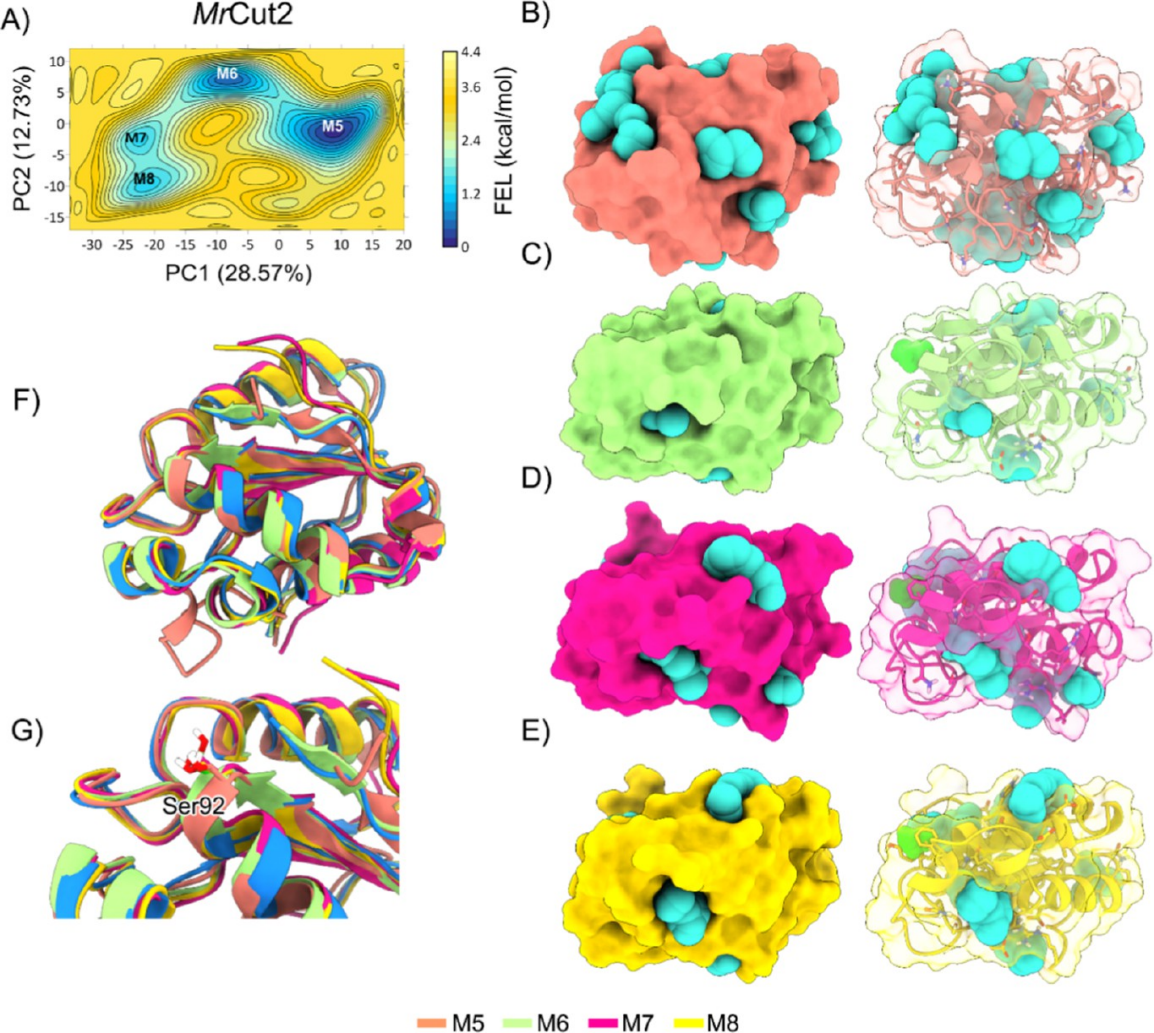
(A) Free energy landscape (FEL) result for *Mr*Cut2, with the energy surface in kcal/mol mapped as a function of the principal components PC1 and PC2. The representative structures of each minimum are shown, highlighting the pocket volumes (in cyan spheres) for each structure. (B) Structure corresponding to minimum M1. (C) Structure corresponding to minimum M2. (D) Structure corresponding to minimum M3. (E) Structure corresponding to minimum M4. (F) Comparison of the structures of minima M1, M2, M3 and M4. (G) Alignment of the regions of the catalytic triad.

The FEL analysis for *Mr*Cut3 identified four energy minima: M9, M10, M11, and M12 (Figure 6A). M9 displays an open conformation with a well-defined pocket in the active site region, which could facilitate substrate recognition and binding, suggesting a potential pathway for polymer degradation (Figure 6B). The other minima, M10, M11, and M12 (Figure 6C–E), correspond to higher energy conformational states with smaller pockets in the active site. These conformations do not represent the ideal open state for catalytic coupling, suggesting that substrate access would be more restricted in these states.

**Figure 6:**
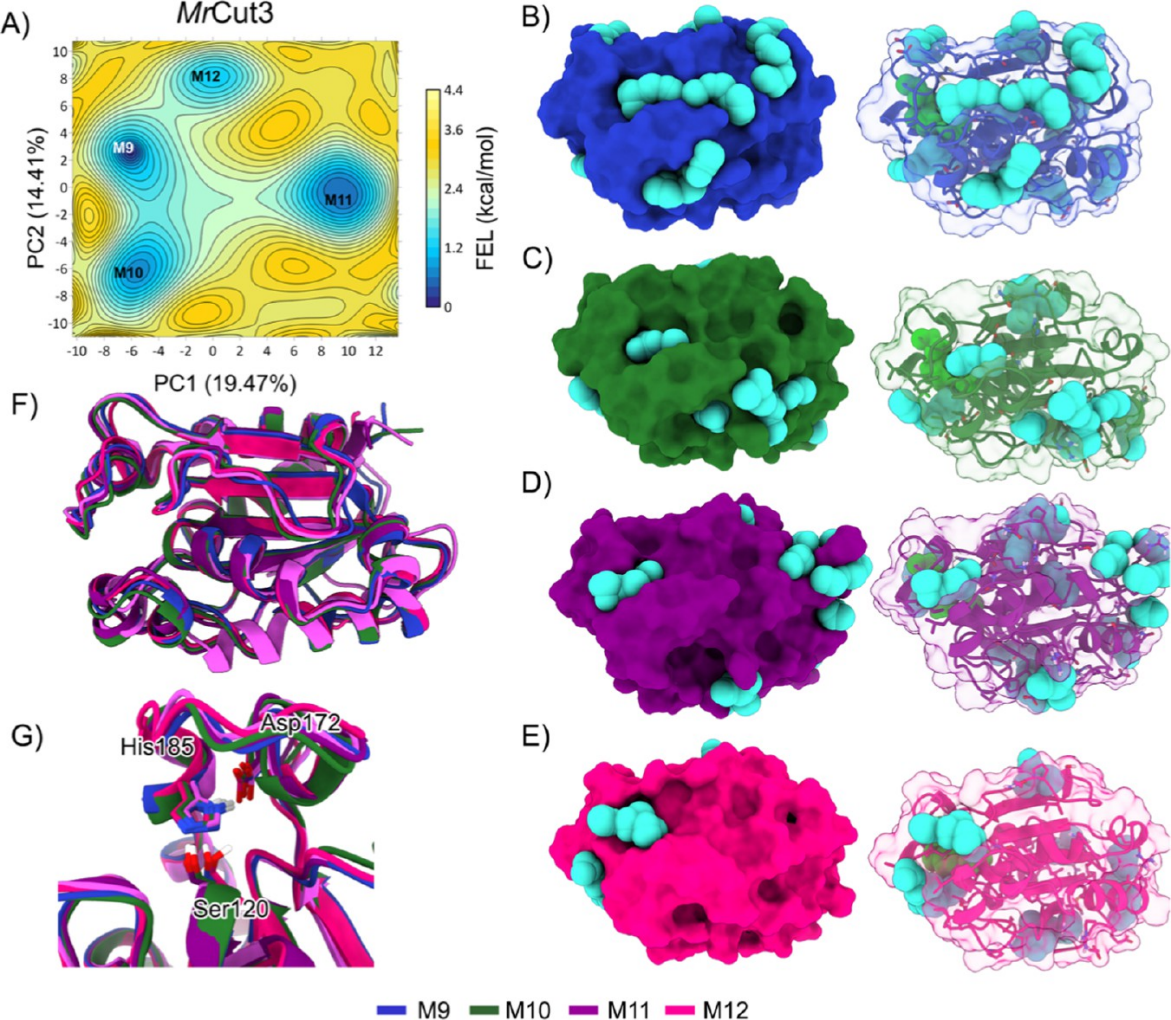
(A) Free energy landscape (FEL) result for *Mr*Cut3, with the energy surface in kcal/mol mapped as a function of the principal components PC1 and PC2. The representative structures of each minimum are shown, highlighting the pocket volumes (in cyan spheres) for each structure. (B) Structure corresponding to minimum M1. (C) Structure corresponding to minimum M2. (D) Structure corresponding to minimum M3. (E) Structure corresponding to minimum M4. (F) Comparison of the structures of minima M1, M2, M3 and M4. (G) Alignment of the catalytic triad regions.

The modifications observed in *Mr*Cut3 are similar to those found in *Mr*Cut1, focusing mainly on the active site region where the catalytic triad Ser92, His157, and Asp144 is located (Figure 6G). When comparing *Mr*Cut1, *Mr*Cut2, and *Mr*Cut3, we observe that *Mr*Cut1 and *Mr*Cut3 exhibit similar patterns of conformational modulation, where molecular dynamics reveals the formation of cavities in the active site at energy minima states, suggesting that these conformations favor interaction with the PET substrate. These modifications are particularly located in the active site region, where increased cavity volume facilitates the fitting of the PET polymer by transitioning the protein from a closed active site state to an open state capable of accommodating the substrate. In contrast, *Mr*Cut2 differs significantly as it does not contain the complete catalytic triad and does not form a sufficiently open cavity in the active site to accommodate the substrate. These characteristics suggest that *Mr*Cut2 cannot play a direct role in PET degradation, although it may have an auxiliary or complementary function in the organism yet to be explored. Thus, our simulations demonstrate that proteins undergo conformational changes over time to increase the active site cavity, allowing for more effective interaction with the substrate and potentially facilitating the degradation process.

Although *Mr*Cut2 lacks a complete catalytic triad and does not present a well-defined active site cavity, our data suggest that this protein may still play a relevant biological role within *M. roreri*. It is plausible that *Mr*Cut2 acts as an accessory protein, potentially contributing to substrate recognition or modulating the expression and/or conformational dynamics of catalytically active cutinases, such as *Mr*Cut1. Alternatively, *Mr*Cut2 may be involved in distinct metabolic processes unrelated to polymer degradation, which remain to be elucidated.

Moreover, the structural integrity and stability observed during MD simulations, along with conserved elements shared with other fungal cutinases, suggest that *Mr*Cut2 retains the structural framework necessary for catalytic function. From an evolutionary standpoint, the reconstitution of the catalytic triad, whether by natural mutation or rational design, could restore or confer enzymatic activity. Such a scenario could allow *Mr*Cut2 to degrade alternative synthetic substrates or polymers not efficiently processed by *Mr*Cut1 or *Mr*Cut3. This hypothesis highlights the importance of future studies focused on site-directed mutagenesis and structural engineering to explore the hidden catalytic potential of *Mr*Cut2.

### Formation of the Cutinase-Polymer Complex

For *Mr*Cut1 and *Mr*Cut3 in the ligand-free form, from the lowest-energy region of the free energy landscape, we identified a representative conformation in which the active-site cavity appeared fully formed and structurally stable. This structure was selected as the reference model for the polymer-placement procedures, ensuring that the subsequent enzyme–polymer simulations were initiated from an energetically favorable state. Both the *Mr*Cut1 and *Mr*Cut3 complexes showed RMSD values below 2.5 Å (Figures S4 and S5), indicating that all five simulation replicates (300 ns each) remained stable. Clustering of all replicates for each system was performed jointly to evaluate the overall trajectory behavior across the full 1.5 μs of simulation time. More than 500 ns of each system showed high frame similarity, yielding a refined trajectory that represents at least 50% of the sampled conformations (Table S2 and Figure S6). Based on this analysis, we used the interval from 200 to 500 ns for the binding free-energy calculations, applying the same 300 ns window to all complexes and ensuring that the selected region corresponded to a shared stability plateau.

To analyze enzyme conformational changes upon ligand binding, we also performed FEL analyses of *Mr*Cut1 and *Mr*Cut3 in complex with PES, PCL, and PET. We begin by discussing the *Mr*Cut1–PES complex. The multimodal profiles observed in the one-dimensional histograms of PC1 and PC2 reveal transitions between structurally distinct conformations over the course of the simulation (see Figure S7). This behavior suggests enhanced structural plasticity of *Mr*Cut1 in the presence of PES. In contrast, the *Mr*Cut1–PCL system exhibits a markedly more compact conformational distribution, concentrated around a dominant cluster with limited dispersion along both principal components. The corresponding PC1 and PC2 histograms show narrower, predominantly unimodal distributions, indicating that the system remains largely confined to a single conformational basin with only local fluctuations. This behavior points to a more restricted dynamical regime when PCL is bound in the active site. The *Mr*Cut1–PET complex represents an intermediate scenario. Although a predominant conformational region is present, the distribution extends continuously along PC1, consistent with the larger proportion of variance explained by this component. The histograms reveal partially overlapping populations, suggesting smoother transitions between conformational states rather than well-separated substates.

PC1 and PC2 explain 27.1% and 21.3%, 18.5% and 16.4%, and 32.2% and 15.5% of the total variance for *Mr*Cut1 in complex with PES, PCL, and PET, respectively (Figure S7). The FEL constructed along PC1 and PC2 further clarify the differences in the conformational behavior of the three complexes. For the *Mr*Cut1–PES system, the surface is clearly characterized by multiple well-defined minima (M1-M4), separated by noticeable energy barriers (Figure 7). M1 corresponds to the global minimum, while M2, M3, and M4 represent additional energetically accessible states. The presence of four distinct regions indicates a rugged energy landscape, consistent with the broad and fragmented distribution observed in the PCA. This pattern suggests that PES binding allows the enzyme to occupy multiple metastable conformational states, rather than remaining confined to a single dominant region.

**Figure 7:**
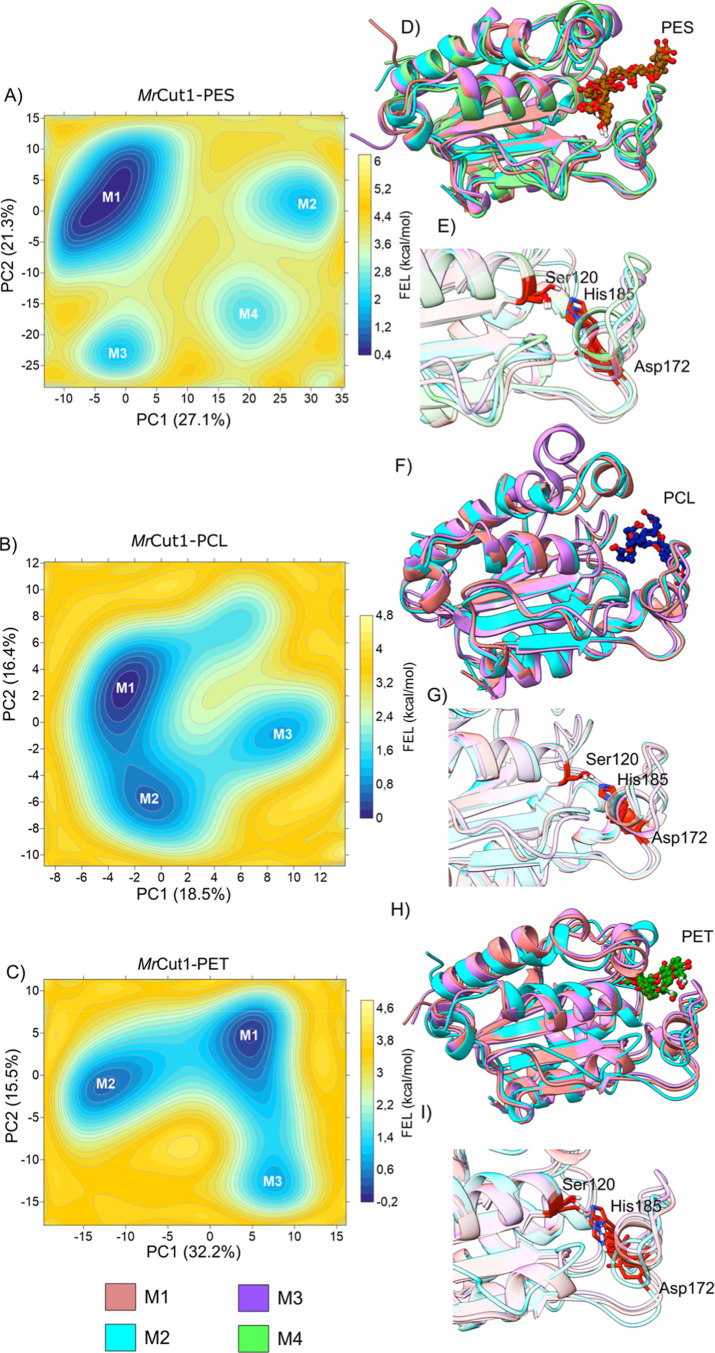
Free energy landscape (FEL) result for *Mr*Cut1 (A–C), with the energy surface in kcal/mol mapped as a function of the principal components PC1 and PC2. The representative structures of each minimum are shown. (D,F,H) All Structure corresponding to minima with each polymer in the active site. (E,G,I) Alignment of the catalytic triad regions for each polymer complex, PES, PCL and PET, respectively.

In contrast, the *Mr*Cut1–PCL complex exhibits a smoother and less fragmented surface. Although three minima regions (M1-M3) can be identified, M1 is clearly predominant and the surrounding basins appear more connected, with reduced energetic separation compared to *Mr*Cut1–PES complex. The energy differences between these states are smaller, and the transitions between them appear more continuous. For the *Mr*Cut1–PET complex, the FEL reveals an intermediate scenario. Three minima regions are observed (M1-M3), with M1 and M2 being energetically relevant, while M3 appears shallower. The basins are distinct but connected by relatively continuous low-energy regions, showing accessible transitions between conformations. Unlike the *Mr*Cut1–PES complex, the energy surface does not exhibit highly isolated minima, although it is more heterogeneous than the *Mr*Cut1–PCL complex. This indicates moderate conformational flexibility induced by the PET.

The conformational space explored by *Mr*Cut3 differs markedly depending on the bound polymer, as evidenced by the projections along the first two principal components (Figure S8). The relative contribution of these components to the total variance is 30.8% (PC1) and 26.5% (PC2) for the PES complex, 26.9% and 12.8% for PCL, and 44.6% and 9.7% for PET. For the *Mr*Cut3–PES system the sampled points are organized into multiple populated domains with notable separation between them. This pattern indicates that the enzyme does not fluctuate around a single dominant conformation, but rather interconverts between structurally differentiated states. In *Mr*Cut3–PCL, the distribution is considerably more localized (Figure S8). The conformational cloud is centered around a primary region with limited dispersion, particularly along PC2, which contributes a smaller fraction of the total variance. The reduced dispersion indicates that most fluctuations correspond to small-amplitude movements around a dominant structural state. One dimensional projections reinforce this observation, as the density is concentrated within a narrower range of values (Figure S8).

The *Mr*Cut3–PET complex exhibits distinct behavior, characterized by strong elongation along PC1. With nearly half of the total variance explained by this component, structural variability is largely governed by a single dominant collective movement. Conformational points extend continuously along this axis, while remaining comparatively compressed along PC2. The distribution along PC1 suggests the presence of multiple accessible configurations that are not sharply segregated but rather connected through gradual structural transitions. This indicates directional conformational flexibility, in which the enzyme undergoes progressive rearrangements along a primary dynamic coordinate, rather than exploring multiple orthogonal substates.

The FEL constructed along PC1 and PC2 further clarify how the polymer nature modulates the conformational energy profile of *Mr*Cut3 (Figure 8), consistently reinforcing the datas observed in the PCA analysis. For the *Mr*Cut3–PES system, the surface is clearly characterized by four well-defined minima (M1-M4), separated by considerable energy barriers. M1 corresponds to the global minimum, while M2, M3, and M4 represent additional energetically accessible states. The presence of four distinct regions indicates a rugged energy landscape, suggesting that PES binding allows the enzyme to occupy multiple metastable conformational states, rather than remaining confined to a single dominant basin.

**Figure 8:**
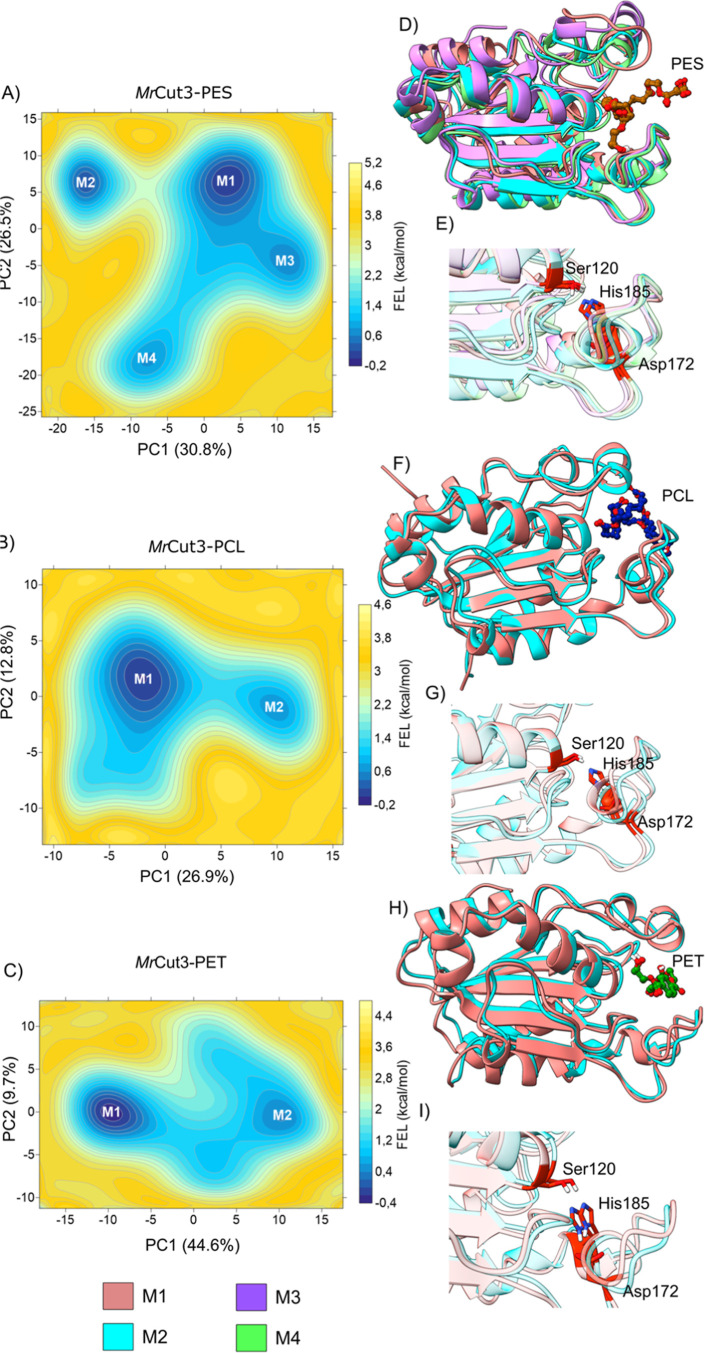
(A) Free Energy Landscape (FEL) result for *Mr*Cut3 (A–C), with the energy surface in kcal/mol mapped as a function of the principal components PC1 and PC2. The representative structures of each minimum are shown. (D,F,H) All Structure corresponding to minima with each polymer in the active site. (E,G,I) Alignment of the catalytic triad regions for each polymer complex, PES, PCL and PET, respectively.

In contrast, the *Mr*Cut3–PCL complex exhibits a smoother and less fragmented surface. Two main minima (M1 and M2) can be identified, with M1 clearly predominant and the basins appearing more connected, showing reduced energy separation compared to the potential energy surface (PES). The energy differences between these states are smaller, and the transitions between them appear more continuous. This behavior indicates that PCL stabilizes a more restricted conformational set. For the *Mr*Cut3–PET complex, the FEL reveals an intermediate scenario. Two relevant minima (M1 and M2) are observed, with M1 being the more stable state. The basins are distinct but connected by relatively continuous low-energy regions, indicating accessible conformational transitions. Unlike the PES system, the energy surface does not exhibit highly isolated minima, although it remains more heterogeneous than that observed for PCL. This suggests moderate conformational flexibility induced by PET binding.

Experimental data show that the synthetic polymers PES, PCL, and PET were biodegraded by *Mr*Cut1, with approximately 59%, 43%, and 31% degradation, respectively.^32^ Although biodegradation assays have not yet been conducted for *Mr*Cut3, in silico results indicate that this enzyme may also degrade PES, PCL, and PET, with Δ*G* values of −7.99 ± 0.43, −7.30 ± 0.42, and −7.95 ± 0.60 kcal/mol, respectively (Table 2), as estimated by the SIE method. The high structural similarity between *Mr*Cut1 and *Mr*Cut3 supports this hypothesis. In particular, the resemblance in their active sites and the similar energy minima observed in the Free Energy Landscape (FEL) analysis reinforce the likelihood that both enzymes can degrade synthetic polymers.

**Table 2:** Free Energy Values, in kcal/mol, Obtained Using the SIE Method for the Enzymes *Mr*Cut1 and *Mr*Cut3 Complexed with the Polymers PES, PCL, and PET

SIE			
*Mr*Cut1			
energy (kcal/mol)	polymer		
	PES	PCL	PET
**Inter vdW**	–54.84 ± 4.00	–50.70 ± 4.33	–47.65 ± 3.71
**Inter Coulomb**	–6.20 ± 4.39	–15.20 ± 4.76	–7.72 ± 3.23
**Reaction Field**	11.64 ± 3.98	18.23 ± 3.47	11.34 ± 1.61
**Cavity**	–10.53 ± 0.82	–10.63 ± 0.63	–8.54 ± 0.53
**Constant**	–2.89	–2.89	–2.89
**DeltaG**	–9.17 ± 0.49	–9.00 ± 0.56	–8.40 ± 0.49
*Mr*Cut3			
Inter vdW	–48.06 ± 3.59	–40.19 ± 3.28	–46.02 ± 4.65
Inter Coulomb	–19.38 ± 3.48	29.32 ± 3.34	–3.12 ± 2.60
Reaction Field	–10.82 ± 2.66	–23.19 ± 2.69	9.67 ± 2.09
Cavity	–9.20 ± 0.69	–8.03 ± 0.39	–8.86 ± 0.71
Constant	–2.89	–2.89	–2.89
DeltaG	–7.99 ± 0.43	–7.30 ± 0.42	–7.95 ± 0.60

The main amino acid residues that interact in a common way with the polymers to forms the complexes are Gln45, Pro52, Phe86, Ser120, Gly148, Lys151, Ile174, Val184 and His185, where the catalytic residues Ser120 and His185 stand out and the oxyanion hole residues Thr45 e Gln121 for *Mr*Cut1 and *Mr*Cut3. The interactions involved between the selected pose of each polymer and *Mr*Cut1 are mostly hydrophobic and van der Waals (Table S5). It demonstrates that all 3 polymers were able to locate in the pocket volume of the *Mr*Cut1 active site (M1 structure) and in the same region. For *Mr*Cut3 complex, the main amino acid residues interact with all polymers are Thr46, Phe54, Phe86, Tyr119, Ser120, Val181, His185, highlighting Ser120 and His185 which are catalytic residues. We noticed that the main residues, Tyr, Phe, and Val, are nonpolar and involved in interactions with the hydrophobic chain of the polymers. Furthermore, these nonpolar residues can contribute to the alignment of the molecule in the active site, facilitating the nucleophilic attack of Ser120 on the ester group. We also highlight the polar residues, which, through hydrogen bonding interactions, play a fundamental role in stabilizing the molecule in the active site as in the cutinase enzyme of *F. oxysporum*.^15^ Similar to *Mr*Cut1, complexes with *Mr*Cut3 are primarily stabilized by hydrophobic and van der Waals interactions (Table S6).

Additionally, we monitored hydrogen bonds (HBs) formed between the ligands and the oxyanion hole residues in both the *Mr*Cut1 and *Mr*Cut3 systems. For *Mr*Cut1, the average numbers of hydrogen bonds with Gln121 and Thr43, calculated over the five replicates, were 2.20 ± 0.34 and 2.13 ± 0.20 for the PES complex, 5.30 ± 0.35 and 6.63 ± 0.31 for the PCL complex, and 4.86 ± 0.99 and 2.79 ± 0.96 for the PET complex, respectively. PCL did not form stable hydrogen bonds with both residues simultaneously, whereas PET interacted predominantly with Thr43 only. For *Mr*Cut3, the average hydrogen-bond counts with Gln121 and Thr45 over the simulations were 2.48 ± 0.36 and 2.01 ± 0.18 for PES, 3.01 ± 0.36 and 1.94 ± 0.16 for PCL, and 5.22 ± 0.77 and 4.96 ± 0.63 for PET. These observations are consistent with the available experimental data, which indicate that *Mr*Cut1 degrades PES more efficiently than PCL or PET.^32^ Therefore, the results suggest that the short distance observed in *Mr*Cut1–PES complexes may allow favorable electrostatic interactions with the oxyanion hole, facilitating stabilization of the transition state and catalytic intermediates during the chemical reaction.

These results are also consistent with the experimental data, which show that *Mr*Cut1 degrades PES more effectively than PCL and PET. The binding affinity calculations indicate that PCL presents higher affinity for *Mr*Cut1 and *Mr*Cut3 than PET ligands.

The van der Waals interactions (Figure 9B,C) reveal an important trend, where PES and PCL polymers show stronger interactions than PET, with values (Inter vdW) of −54.84 ± 4.0, −50.70 ± 4.33, and −47.65 ± 3.71 kcal/mol, respectively (Table 2). These results suggest that PES and PCL have molecular structures that favor stronger interactions with apolar and hydrophobic residues of the enzyme compared to the PET polymer, which contains an aromatic ring in its structure and is not similar to the other two polymers (Figure 9B,D). This directly results in lower structural compatibility between the polymer and *Mr*Cut1, which impairs the formation of the *Mr*Cut1–PET complex, as observed through the Fingerprint interaction analysis. This analysis shows that the main vdW interactions with PET are weaker than those with the PES and PCL polymers (Figure 9B). In the vdW interactions involved in the complexes with *Mr*Cut3, PES presents a stronger vdW value (−48.06 ± 3.59 kcal/mol), followed by PET (−46.02 ± 4.65 kcal/mol), and finally PCL (−40.19 ± 3.28 kcal/mol). However, due to amino acid changes in the active site between the different cutinase structures, PET shows a much greater affinity for the active site, which is directly linked to the polymer binding mode at the site (Figure 9C,E).

**Figure 9:**
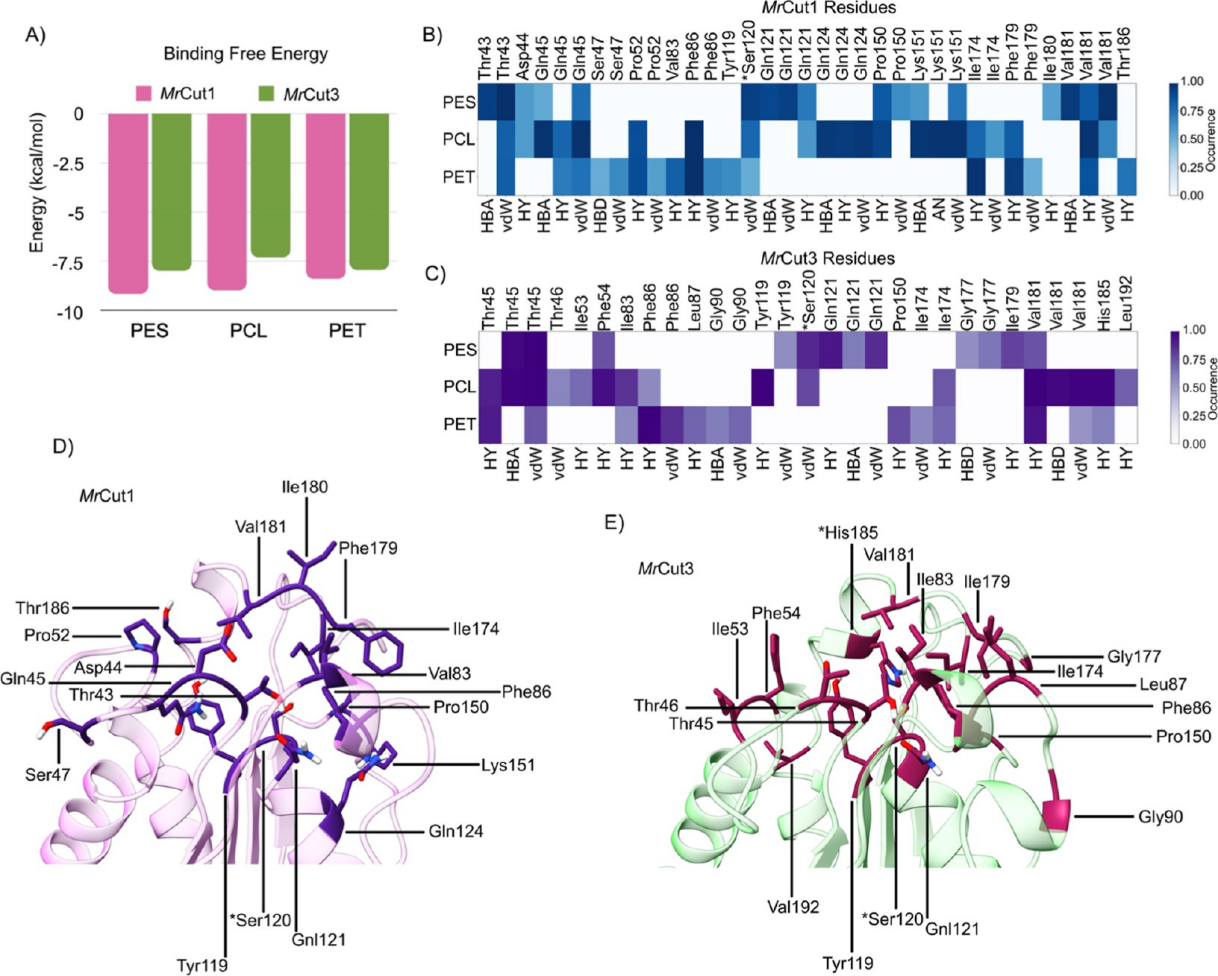
Analysis of the chemical and energetic contributions involved in the formation process of the enzyme–polymer complex. (A) Binding free energy Δ*G* values (in kcal/mol) obtained through SIE method for *Mr*Cut1 and *Mr*Cut3. Analysis of the fingerprint interactions in the formation of the complex involving the polymers PES, PCL, and PET with the enzyme (B) *Mr*Cut1 and (C) *Mr*Cut3, highlighting the active site residues involved in these interactions in the three-dimensional structure of the enzyme (D) *Mr*Cut1 and (E) *Mr*Cut3.

Coulombic interactions provide insights in the formation of the *Mr*Cut1–polymer complex, with PCL exhibiting the highest electrostatic interaction (−15.20 ± 4.76 kcal/mol), indicating a stronger attraction between the enzyme and the polymer due to the charged groups present in PCL. In contrast, PET and PES show weaker interactions (−6.20 ± 4.39 kcal/mol and −7.72 ± 3.23 kcal/mol, respectively), suggesting lower polarity in these polymers, which results in less pronounced electrostatic interactions with the enzyme (Table 2 and Figure 9).

For the *Mr*Cut3–PCL complex, electrostatic interactions were unfavorable (+29.32 ± 3.34 kcal/mol), indicating that binding is primarily driven by van der Waals forces (−40.19 ± 3.28 kcal/mol), as previously discussed. In contrast, PES showed a favorable electrostatic interaction (−19.38 ± 3.48 kcal/mol), suggesting strong electrostatic attraction that may contribute to the formation of a more stable complex. PET, however, exhibited a much weaker electrostatic interaction (−3.12 ± 2.60 kcal/mol) compared to the other polymers, indicating lower electrostatic contribution and reduced complex stability.

The binding free energy values (Δ*G*) are more negative for the PES polymer (−9.17 ± 0.49 kcal/mol), indicating the polymer with the highest affinity for the *Mr*Cut1 enzyme, followed by PCL (−9.00 ± 0.56 kcal/mol) and PET (−8.40 ± 0.49 kcal/mol) (Figure 9A and Table 2). Therefore, the PES and PCL polymers exhibit stronger interactions with the *Mr*Cut1 enzyme compared to PET, suggesting that *Mr*Cut1 is more efficient at degrading PES and PCL than PET, in agreement with experimental data^32^

For the *Mr*Cut3, PES demonstrates the highest affinity, with a value of −7.99 ± 0.43 kcal/mol, followed by PET (−7.95 ± 0.60 kcal/mol) and PCL (−7.30 ± 0.42 kcal/mol) (Figure 9A). These results indicate that PES exhibits a stronger affinity for the *Mr*Cut3 enzyme, as evidenced by van der Waals interactions, electrostatic interactions, and binding free energy (Table 2). Overall, our findings indicate that hydrophobic and van der Waals interactions are the main driving forces in the formation of Polymer-cutinase complexes studied here (Table 2), with electrostatic contributions playing a variable role depending on the polymer. PES shows the highest affinity for both enzymes, followed by PCL and PET, correlating well with experimental degradation efficiencies. These findings highlight the importance of polymer-specific interactions in determining enzymatic binding and provide valuable insight into the molecular determinants underlying cutinase-mediated polymer degradation, offering guidance for the rational design of more efficient biocatalysts.

## Conclusion

In this study, we used computer simulations for understanding the interaction mechanisms of the enzymes *Mr*Cut1 and *Mr*Cut3 with different synthetic polymers: polyethylene succinate (PES), polycaprolactone (PCL), and polyethylene terephthalate (PET). Detailed analysis of the molecular interactions (IFP analysis) and binding mode revealed how the same active site can accommodate structurally distinct polymers, highlighting the catalytic flexibility of these enzymes. Additionally, we demonstrated for the first time that both the *Mr*Cut1 and *Mr*Cut3 enzymes have binding affinity with the polymers PES, PCL, and PET. *Mr*Cut3 exhibits affinity for PES (−7.99 ± 0.43), PET (−7.95 ± 0.60 kcal/mol), and PCL (−7.30 ± 0.42 kcal/mol), whereas *Mr*Cut1 shows affinity for PES (−9.17 ± 0.49 kcal/mol), PCL (−9.00 ± 0.56 kcal/mol), and PET (−8.40 ± 0.49 kcal/mol). Structural analysis demonstrated that the *Mr*Cut1 and *Mr*Cut3 enzymes share more than 50% similarity, highlighting the presence of two disulfide bridges that play a fundamental role in stabilizing the active site and in regulating the opening and closing of the catalytic cavity.

Although *Mr*Cut2 does not exhibit catalytic activity due to the absence of a complete catalytic triad and a well-defined active site cavity, its structural stability and conservation of key regions suggest a potential auxiliary or regulatory role within *M. roreri* *.* Furthermore, considering its preserved cutinase fold, *Mr*Cut2 may regain catalytic functionality through evolutionary processes or protein engineering, expanding its potential as a biocatalyst in future applications. Finally, the FEL analysis, together with site cavity analysis, demonstrated that both the *Mr*Cut1 and *Mr*Cut3 enzymes exhibit similarity in the active site volume throughout the simulation time, maintaining well-defined minimum-energy structures. Furthermore, the data obtained support the future expression of these enzymes in heterologous systems, targeting industrial and environmental applications related to the degradation of polymers.

In addition to these findings, our results show that the same fungus can produce different cutinase isoforms depending on the growth conditions. Although *Mr*Cut1 and *Mr*Cut3 originate from the same organism, they exhibit structural differences that alter the architecture of the active site. As a result, each isoform interacts differently with PES, PCL, and PET, consistent with the affinity values we observed for these polymers. These data indicate that *M. roreri* adjusts its enzymatic profile according to environmental conditions, generating isoforms with specific structural features that influence substrate preference and expand its ability to act on different types of polymers.

## Supplementary Material



## Data Availability

Topology files, inputs, coordinates and three-dimensional structures are available in the GitHub repository https://github.com/carlos-desouza/MrCut-polymer-paper. All simulations were performed using the AMBER package, available at https://ambermd.org.

## Notes

FASTA sequences and GenBank accession numbers of the *M. roreri* cutinases investigated, clustering analyses of *Mr*Cut1 and *Mr*Cut3 polymer–enzyme complexes, and structural validation metrics based on Ramachandran statistics. Additional data include three-dimensional structural models and comparisons with *Is*PETase, principal component analysis (PCA) of the conformational space sampled during the simulations, RMSD profiles obtained from the five independent replicas of each enzyme–polymer system, which were used to define the time window for binding free energy calculations, active site volume analysis obtained with Fpocket, and detailed interaction fingerprints between polymers and active site residues for *Mr*Cut1 and *Mr*Cut3 complexes (PDF)
