# Supplementary material for: Molecular Dynamics Insights into the Biodegradation of Synthetic Polymers by Moniliophthora roreri Cutinases
Source: J Chem Inf Model. 2026 Mar 24;66(7):4085–100. doi: 10.1021/acs.jcim.5c03051 (PMC13081001; doi:10.1021/acs.jcim.5c03051)
Supplement: Supplementary file 1 [file ci5c03051_si_001.pdf]

SUPPORT INFORMATION:

Molecular Dynamics Insights into the Biodegradation of Synthetic Polymers by *Moniliophthora roreri* Cutinases

Maycon Vinicius Damasceno de Oliveira[1], Gabriel Calandrini[2], Carlos Gabriel da Silva de Souza[1], Clauber H S da Costa[3], Munir S Skaf[3] and Jerônimo Lameira[1*]

[1] Laboratório de Planejamento e Desenvolvimento de Fármacos, Instituto de Ciências Exatas e Naturais, Universidade Federal do Pará, 66075-110, Belém, Pará, Brasil.

[2] Programa de Pós-Graduação em Ecologia Aquática e Pesca (PPGEAP), Universidade Federal do Pará, 66075-110, Belém, Pará, Brasil.

[3] Institute of Chemistry and Center for Computing in Engineering & Sciences, University of Campinas – UNICAMP. Campinas, SP 13084-862, Brazil.

**Corresponding Authors: JL, lameira@ufpa.br,*

Maycon Vinicius Damasceno de Oliveira: 0000-0003-2263-2124.

Gabriel Calandrini: 0000-0001-6321-2228.

Carlos Gabriel da Silva de Souza: 0000-0001-6572-7196.

Clauber Henrique Souza da Costa: 0000-0002-6915-1056.

Munir S Skaf: 0000-0001-7485-1228.

Jerônimo Lameira: 0000-0001-7270-1517.

CONTENTS

Supplementary Text

Text S1 .... *S4*
Text S2 .... *S4*
Text S3 .... *S5*
Table S1 .... *S7*
Table S2 .... *S7*
Table S3 .... *S8*
Table S4 .... *S8*
Table S5 .... *S9*
Table S6 .... *S10*
Figure S1 .... *S12*
Figure S2 .... *S13*
Figure S3 .... *S14*
Figure S4 .... *S15*
Figure S5 .... *S16*
Figure S6 .... *S17*
Figure S7 .... *S18*
Figure S8 .... *S19*
Ref. S1 .... *S20*
Ref. S2 .... *S20*
Ref. S3 .... *S20*
Ref. S4 .... *S20*
Ref. S5 .... *S20*
Ref. S6 .... *S20*
Ref. S7 .... *S20*
Ref. S8 .... *S20*
Ref. S9 .... *S20*
Ref. S10 .... *S20*
Ref. S11 .... *S21*
Ref. S12 .... *S21*
Ref. S13 .... *S21*
Ref. S14 .... *S21*

Ref. S15 ....S21

Text S1. Protocol for Cluster Analysis

Structural clustering analysis was performed using the CPPTRAJ module of AmberTools. To identify the main conformational states sampled during the simulations, we applied the hierarchical agglomerative clustering (HierAgglo) method with average linkage. Frame similarity was assessed using RMSD, considering only the heavy atoms of residues 1-176 (C, N, O, CA, CB). An epsilon value of 3.0 Å was used as the cutoff for cluster separation, and the total number of clusters was limited to three.

We performed joint clustering of the five replicas for each *Mr*Cut1 and *Mr*Cut3 complex to refine the conformational sampling and determine, based on the most populated cluster, the trajectory that best represents the overall behavior of the system. This representative trajectory, corresponding to the predominant cluster, was subsequently used for the free energy calculations, ensuring that the analysis was carried out on the most statistically relevant conformational state.

Text S2. Protocol for Binding Free Energy calculations

Binding free energy calculations were performed using 30,000 frames of the MD trajectories, referring to the 200-500ns interval of the trajectory of the most populous cluster in the total trajectory of all replicates each complex (Table S2 and Figure S6) in the SIE program[Ref. S1]. The SIE method employs an implicit solvation model to estimate the binding free energy of a protein-ligand complex in aqueous solution. This $\Delta G_{binding}$ value is obtained by combining the contributions from the interaction energy and the solvation energy [Ref. S1,2]. This value follows the formalism (1):

$$\Delta G_{binding} \approx E_{inter} + \Delta G_{desolv}$$
$$= \left(E_{inter}^{Coul} + \Delta G_{desolv}^{R}\right)_{electrostatic} + \left(E_{inter}^{vdW} + \Delta G_{desolv}^{np}\right)_{non-polar}$$

(1)

Where the $\Delta G_{binding}$ can be described by the contributions of the electrostatic and non-polar components. E_{inter}^{Coul} corresponds to the electrostatic (Coulombic) interaction term. ΔG_{desolv}^{R} denotes the polar component of the electrostatic desolvation free energy. E_{inter}^{vdW} accounts for the van der Waals interactions, while

ΔG_{desolv}^{np} refers to the non-polar contribution to the desolvation free energy [Ref. S1–3].

Text S3. Protocol for performing PCA and FEL analysis

Principal Component Analysis (PCA) and Free Energy Landscape (FEL) analyses are essential for characterizing the conformational dynamics of proteins during MD simulations. In this study, PCA was carried out using the CPPTRAJ module of the Amber22 package, based on the Cα coordinates of the amino acids throughout the simulation. The covariance matrix of these coordinates was diagonalized to obtain the principal components, represented as eigenvectors ordered by their corresponding eigenvalues. PC1 describes the direction of greatest conformational variance, capturing the dominant collective motion sampled during the trajectory. These components enable the visualization and interpretation of the major structural fluctuations and the identification of relevant conformational states that may influence protein function.

The construction of the Free Energy Landscape (FEL) based on the principal components (PCs) enables the calculation of Gibbs free energy and the identification of minimum-energy structures sampled throughout the MD simulations[Ref. S4,5]. These structures, corresponding to the lowest energy states, are defined as those with the highest probability and stability, while transition structures, with higher energies, are less stable and less likely to be found in the minimum energy wells[Ref. S4,6]. FEL operates by obtaining conformations through a statistical description of a protein's energy surface. This approach assumes that conformational transitions occur through the organization of an ensemble of high-probability structures, interconnected by energetically favorable and thermodynamically stable states that converge toward the protein’s native conformation[Ref. S4,7–9]. During MD, the more stable conformations are found in the free energy wells, representing states close to the native, which are essential for the protein's enzymatic function. Thus, FEL provides a detailed view of the most stable conformations and transition states, allowing an in-depth analysis of protein conformational properties [Ref. S10–13].

In the construction of the FEL, the probability distributions along the first two principal components (PC1 and PC2) were converted into free energy values using the Boltzmann relation. It is important to note that this type of analysis

assumes that the conformational ensemble sampled during the MD simulations is sufficiently ergodic and that the system undergoes multiple transitions between meta-stable states. When sampling is limited or transitions are infrequent, the reconstructed free energy surface may be impacted by hysteresis, leading to free energy barriers that are not fully reliable in quantitative terms. For this reason, in the present study the FEL were interpreted only qualitatively, with the purpose of identifying the major conformational basins sampled by each system, rather than extracting absolute barrier heights.

Subsequently, water molecules and counterions were removed from the representative FEL minimum structures. Pocket detection was performed using the open-source Fpocket[Ref.S14] platform, which applies a Voronoi-based algorithm to identify and characterize ligand-accessible cavities, providing pocket descriptors and the corresponding surrounding residues. The spatial relationship, connectivity, and potential communication among pockets located near the catalytic triad were then examined through detailed three-dimensional visual inspection in Chimera, and the pocket volumes were quantified by summing the values obtained from the Fpocket analysis.

The pocket volumes were calculated for the representative structures of the free-energy minima previously identified through PCA and Free Energy Landscape (FEL) analyses. The representative conformations were extracted and processed using cpptraj (AmberTools) and subsequently saved in PDB format. Each structure was then individually analyzed with Fpocket, which detects and characterizes cavities, particularly those associated with the active site [Ref. S15]. The resulting pocket volume measurements are provided in the Supporting Information.

Table S1. FASTA sequence and GenBank accession codes of the *Moniliophthora roreri* cutinases studied in this research.

Protein	Genbank ID	Sequence (FASTA)
***Mr*Cut1**	**ESK97883.1**	MVRVFGLTLLALLVPALAAPVPEDLEARQSGCADVMVVYARGTDQDSPIGD<br>PASVGVLFRDNIKSLLGSR<br>TFSFQGVNYAANVIGFLQGGDPAGSRQMTTDLTNVANSCPNAKIVSAGYSQ<br>GGQLVHNSAAQLTAAVRNR<br>INAVVIFGDPKSDQAVTGIPSSNVKIICHDGDNICEGGFIVTSQHTNYQQDAP<br>AAAQFVLSKV
***Mr*Cut2**	**ESK95146.1**	MAPVERRATVQCADVMVFFARGTTEPAPIGTIVGPPLKAALQRELGSQTMS<br>FQGVDYSANVAGFLQGGDK<br>QGSRTMADDITNAANSCPNAKIVTAGYSQGGQLVHNSAELLSPDVVSRINA<br>AVIFVG
***Mr*Cut3**	**ESK92300.1**	MFTSVARLVLLLGILTPAFAAPLAERAECAEVMVIFARGTTETPPIGIFVGPP<br>FEDALVSQLGGRSLSFQ<br>GVDYAADIAGFLAGGDPAGSRQMAIDITNTANACPNARIVSSGYSQGGQLV<br>HNSAALLSRDIAARINAVV<br>IFGDPKRGQPISGVDSSKVKVFCHDGDNICDGGILVLPPHLNYVVNVVDAA<br>KFVVSKL

Table S2. Results obtained from clustering for both *Mr*Cut1 and *Mr*Cut3 enzyme complexes: cluster ID, centroid, number of frames, and size of cluster as a fraction of the total trajectory (Frac %).

Complex		Cluster ID	Centroid	Number of Frames	Frac (%)
**Protein**	**Polymer**				
***Mr*Cut1**	**PES**	0	1751	126327	84.2
	**PCL**	0	132060	101961	68.0
	**PET**	0	130884	94107	62.7
***Mr*Cut3**	**PES**	0	64400	114078	76.1
	**PCL**	0	93036	83883	55.9
	**PET**	0	91649	93970	62.6

Table S3. Ramachandran values, in percentage, for each of the three-dimensional structures of each Cutinase obtained through the Swiss-Model server.

	Cutinase		
**Ramachandran value (%)**	***Mr*Cut1**	***Mr*Cut2**	***Mr*Cut3**
Swiss-Model	96.10	94.44	96.08
Ramachandran	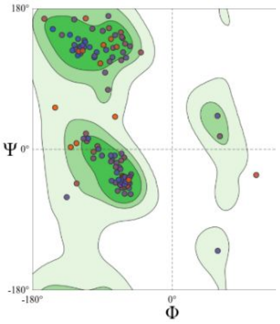	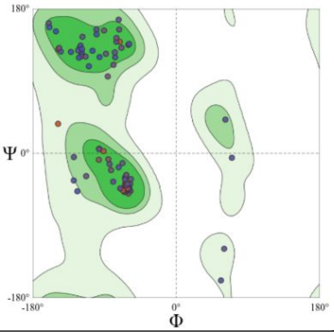	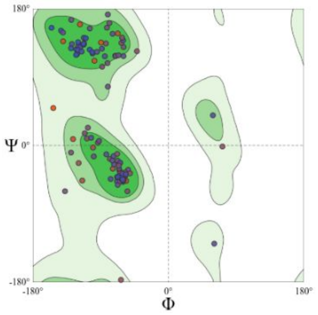

Table S4. Active site volume data obtained using Fpocket.

Enzyme	Identifier	Volume Å^3
	M1	1068.0
***Mr*Cut1**	M2	814.8
	M3	349.9
	M4	482.7
	M5	464.7
***Mr*Cut2**	M6	0
	M7	0
	M8	0
	M9	629.9
***Mr*Cut3**	M10	483.4
	M11	379.7
	M12	391.5

Table S5. Interactions fingerprint between the selected pose of each polymer and amino acid residues from the active site of the *Mr*Cut1 protein for complex formation.

***Mr*Cut1**					
**PES**		**PCL**		**PET**	
**Residue**	**Interaction**	**Residue**	**Interaction**	**Residue**	**Interaction**
Gly15	vdW	Thr16	vdW	Gly15	vdW
Thr16	vdW	Asp17	HY	Thr16	vdW
Asp17	HY		vdW	Asp17	HY
	vdW	Gln18	HY		vdW
Gln18	vdW		vdW	Gln18	HY
Pro25	HY	Pro25	HY		vdW
	vdw		vdW	Ser20	HBD
Phe59	HY	Ala26	HY		vdW
	vdW	Phe59	HY	Pro25	HY
Ser93	vdW		vdW		vdW
Gln94	vdW	*Ser93	vdW	Ala26	vdW
Gly121	vdW	Gln94	HY	Phe59	HY
Asp122	vdW		vdW		vdW
Pro123	vdW	Gly121	vdW	*Ser93	vdW
Lys124	vdW	Asp122	HY	Gly121	vdW
Ile147	HY		vdW	Lys124	HBA
	vdW	Pro123	HY		vdW
Ile153	HY		vdW	Ile147	HY
	vdW	Lys124	HY		vdW
Val154	HY		HBA	Gly150	vdW
	vdW		AN	Gly151	vdW
*His158	HY		vdW	Phe152	HY
	vdW	Ile147	HY		vdW
			vdW	Val154	HY
		Gly150	vdW		vdW
		Val154	HY	*His158	HY
			vdW		vdW
		Thr155	vdW		

		Ser156	vdW		
		*His158	HY		
			vdW		
		Thr159	vdW		

Table S6. Interactions fingerprint between the selected pose of each polymer and amino acid residues from the active site of the *Mr*Cut3 protein for complex formation.

***Mr*Cut3**					
**PES**		**PCL**		**PET**	
**Residue**	**Interaction**	**Residue**	**Interaction**	**Residue**	**Interaction**
Thr46	vdW	Gly44	vdW	Thr45	HY
Phe54	vdW	Thr45	HY		HBA
Ile83	HY		HBA		vdW
	vdW		vdW	Thr46	vdW
Phe86	HY	Thr46	vdW	Glu47	vdW
	vdW	Glu47	HY	Phe54	vdW
Leu87	vdW	Ile53	HY	Phe86	HY
Tyr119	vdW		vdW		vdW
*Ser120	vdW	Phe54	HY	Tyr119	HY
Gln121	HY		vdW		vdW
	vdW	Ile83	HY	*Ser120	vdW
Gly148	vdW		vdw	Gln124	vdW
Asp149	vdW	Phe86	HY	Pro150	HY
Pro150	HY		vdW		vdW
	vdW	Tyr119	HY	Lys151	vdW
Lys151	vdW		vdW	Ile174	HY
Arg152	vdW	*Ser120	vdW		vdW
Ile174	HY	Val181	HY	Gly177	vdW
	vdW		vdW	Gly178	vdW
Gly177	vdW	Pro183	vdW	Ile179	HY
Ile179	vdW	*His185	HY		vdW
Val181	HY	Leu186	HY	Val181	HY
*His185	HY		vdW		vdW

vdW		*His185	HY<br>vdW

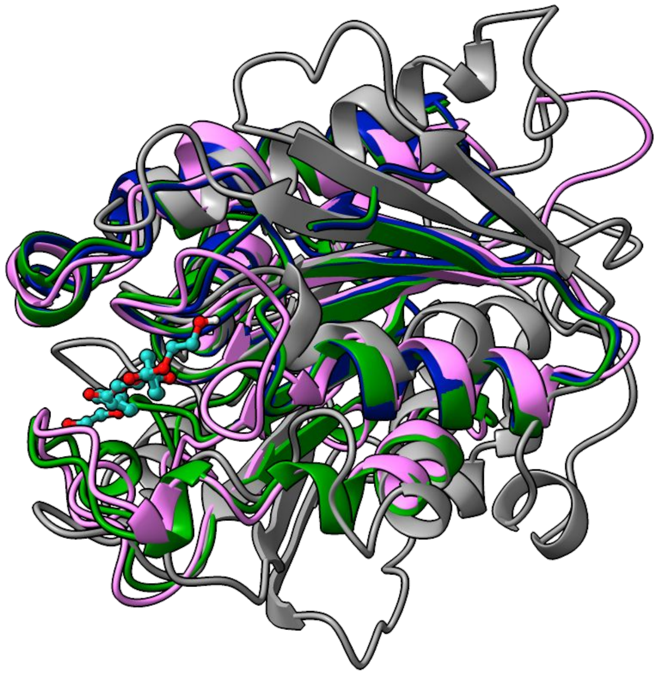

Figure S1. Three-dimensional structures of A) *Mr*Cut1 (pink), B) *Mr*Cut2 (blue), and C) *Mr*Cut3 (green) obtained through the Swiss-Model server. Panel D shows IsPETase (gray) and panel F shows a PET dimer (cyan) for comparison.

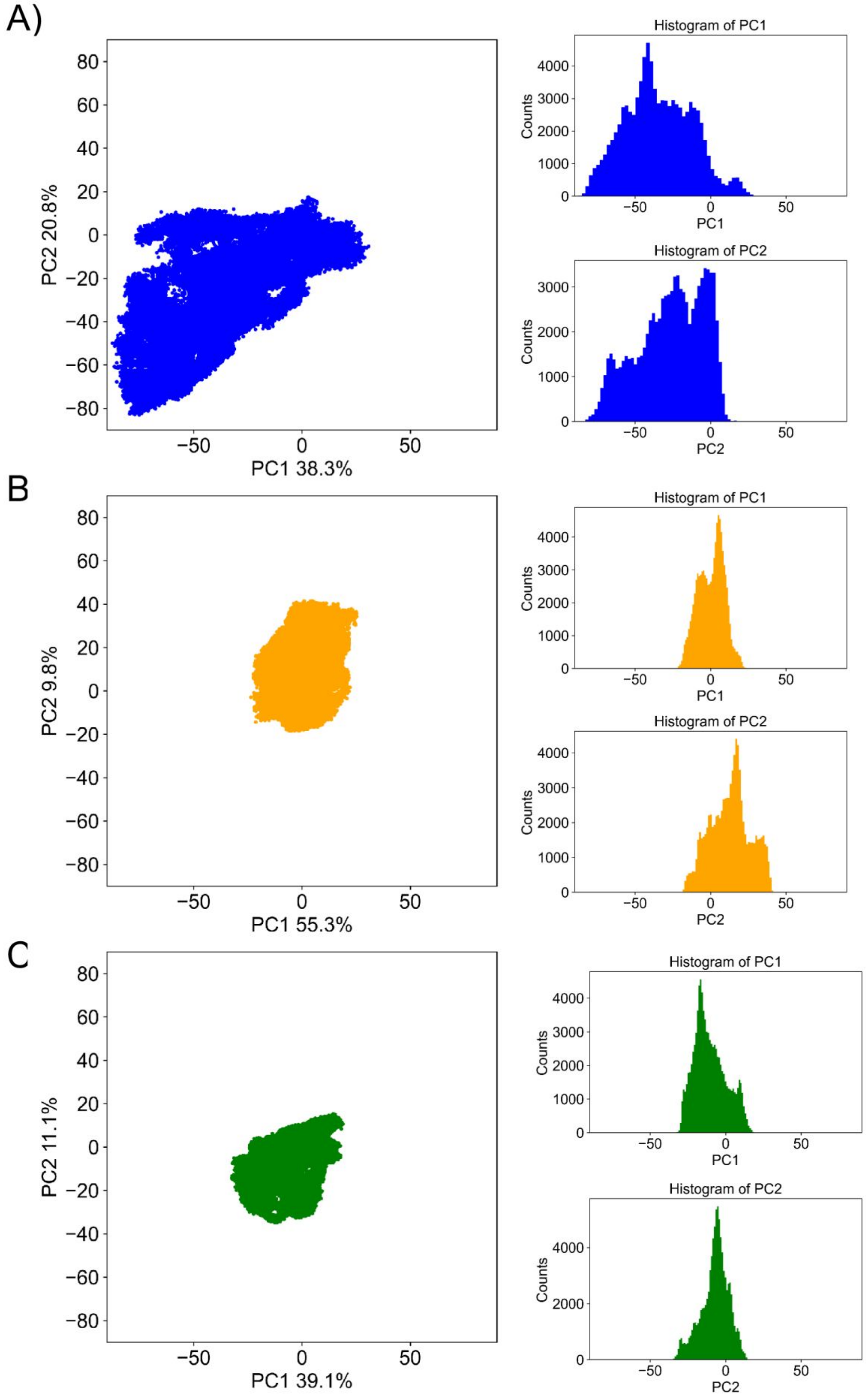

Figure S2. PCA projections (PC1 vs PC2) and 1D histograms of the first two principal components for systems A)*Mr*Cut1, B)*Mr*Cut2, and C)*Mr*Cut3, highlighting differences in the degree of conformational heterogeneity among the variants.

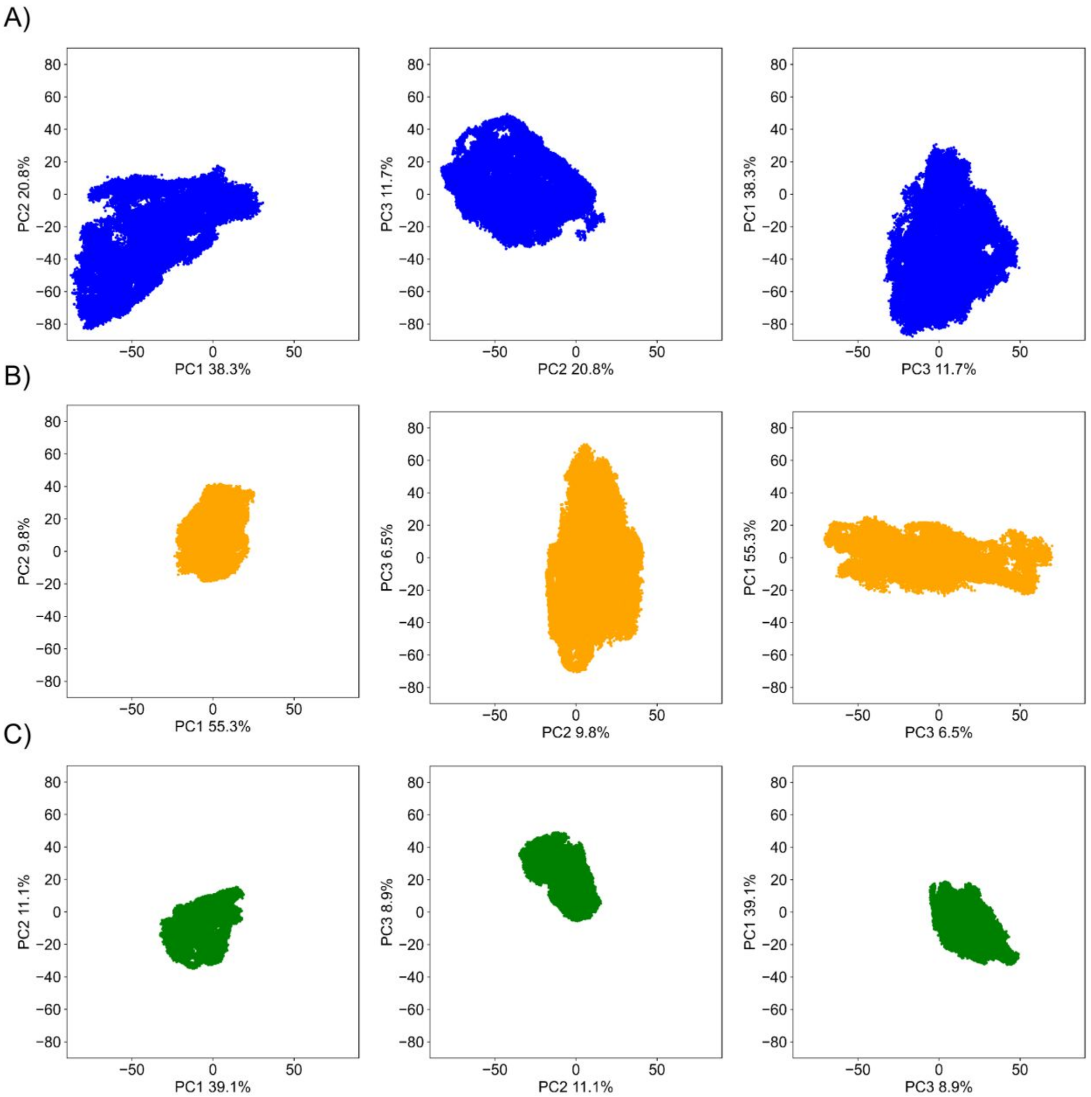

Figure S3. PCA projections for the first three principal components of systems A)*Mr*Cut1, B)*Mr*Cut2, and C)*Mr*Cut3, highlighting the differences in the conformational space explored by each variant throughout the simulations.

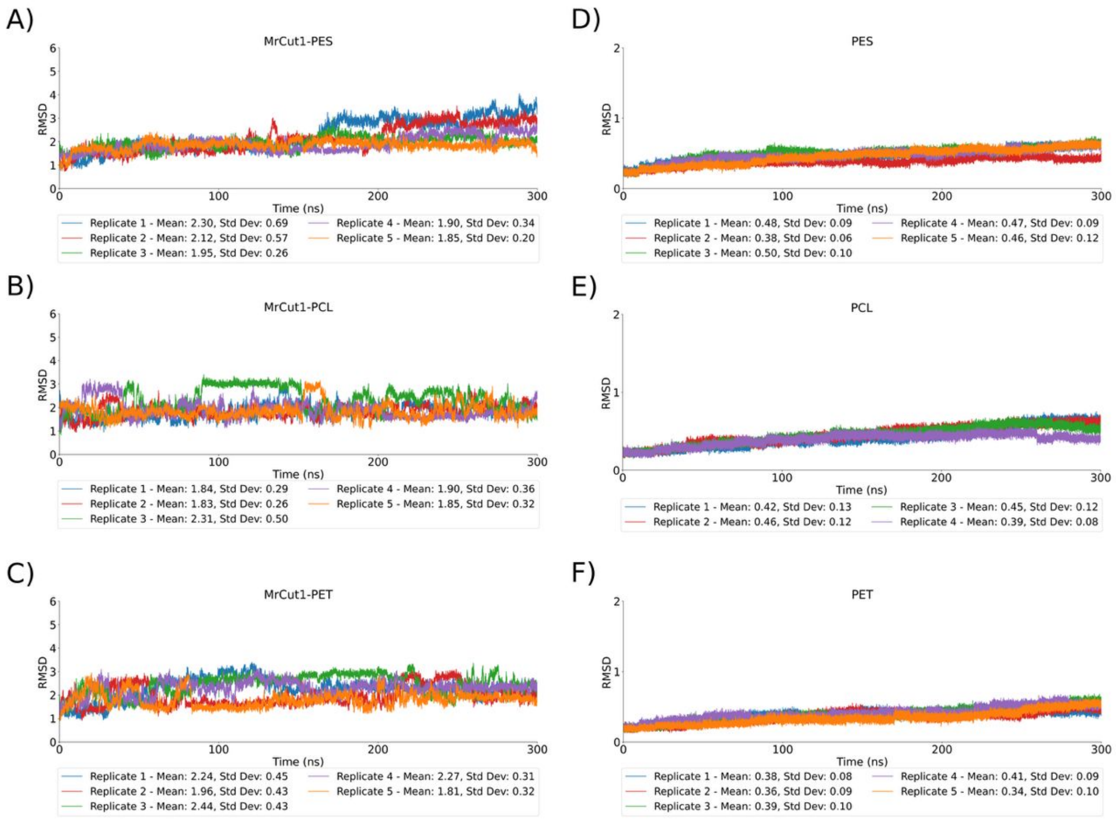

Figure S4. Root-mean-square deviation (RMSD) profiles obtained from five independent molecular dynamics replicas for the enzyme–polymer systems. Panels A–C show the RMSD of the *Mr*Cut1 backbone in complex with PES (A), PCL (B), and PET (C), while panels D–F show the RMSD of the corresponding polymers PES (D), PCL (E), and PET (F) along the simulations.

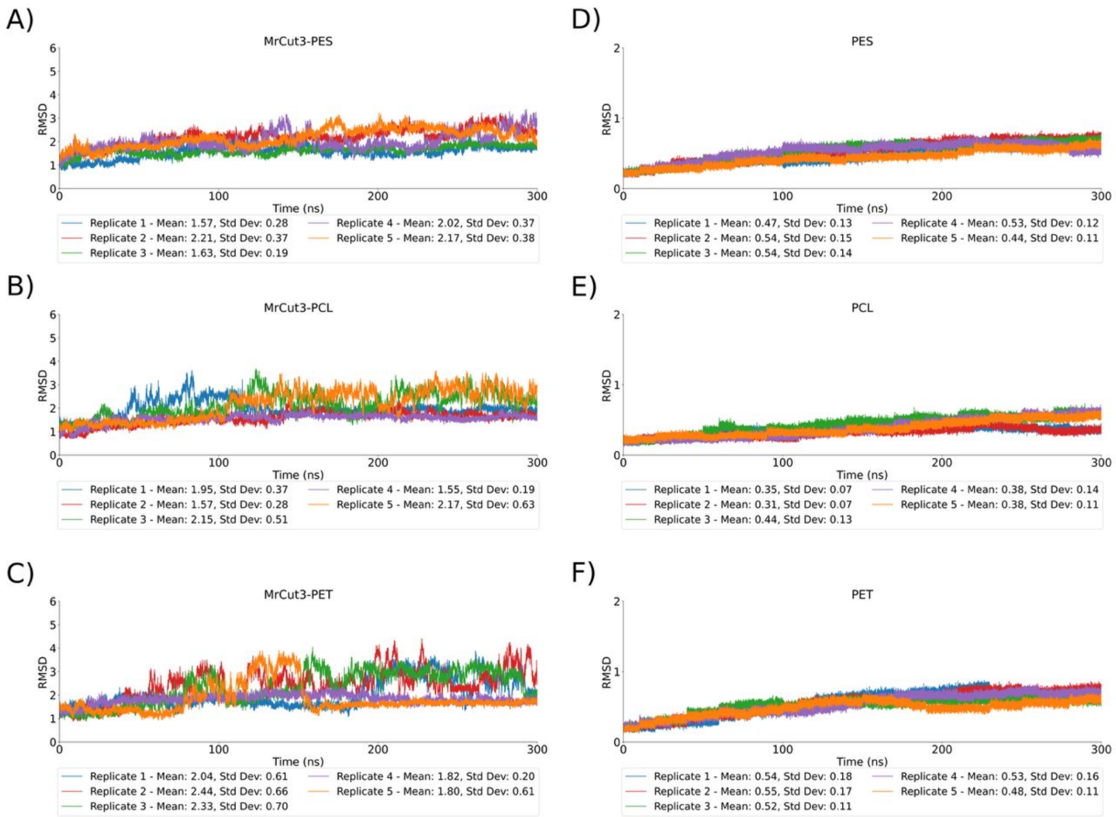

Figure S5. Root-mean-square deviation (RMSD) profiles obtained from five independent molecular dynamics replicas for the enzyme–polymer systems. Panels A–C show the RMSD of the *Mr*Cut3 backbone in complex with PES (A), PCL (B), and PET (C), while panels D–F show the RMSD of the corresponding polymers PES (D), PCL (E), and PET (F) along the simulations.

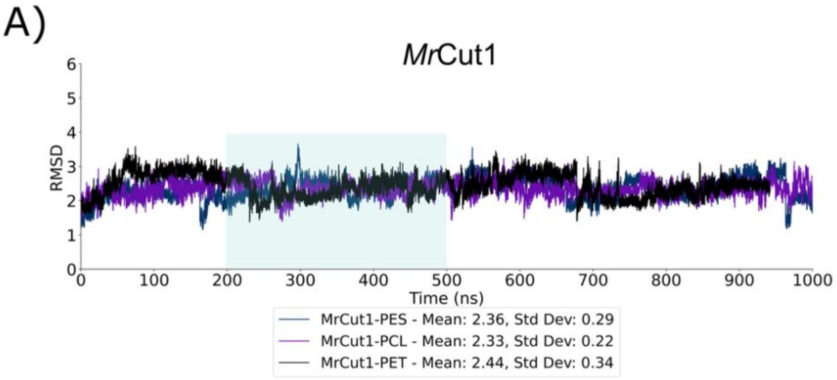

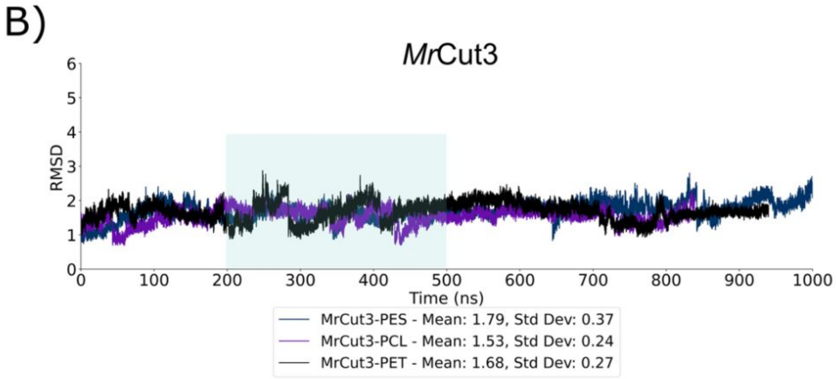

Figure S6. RSMD plot of the trajectories obtained through the set of frames of the selected centroid (Table S2) for each complex of A) *Mr*Cut1 and B) *Mr*Cut3. Highlighting the region of 200 - 500 ns where we performed the binding free energy (SIE) calculation.

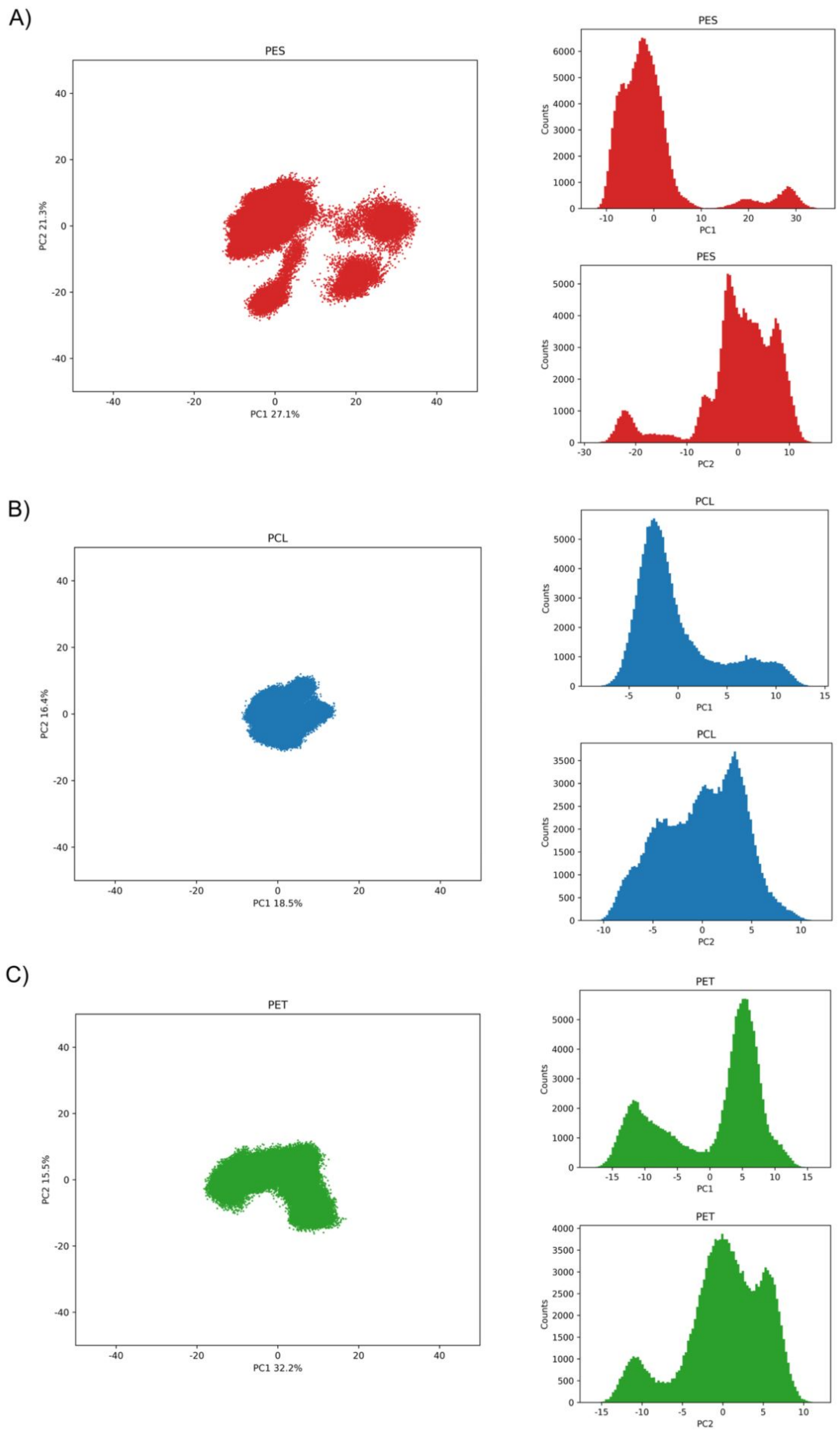

Figure S7. PCA projections (PC1 vs PC2) and 1D histograms of the first two principal components for complexes *Mr*Cut1 with A)PES, B)PCL, and C)PET.

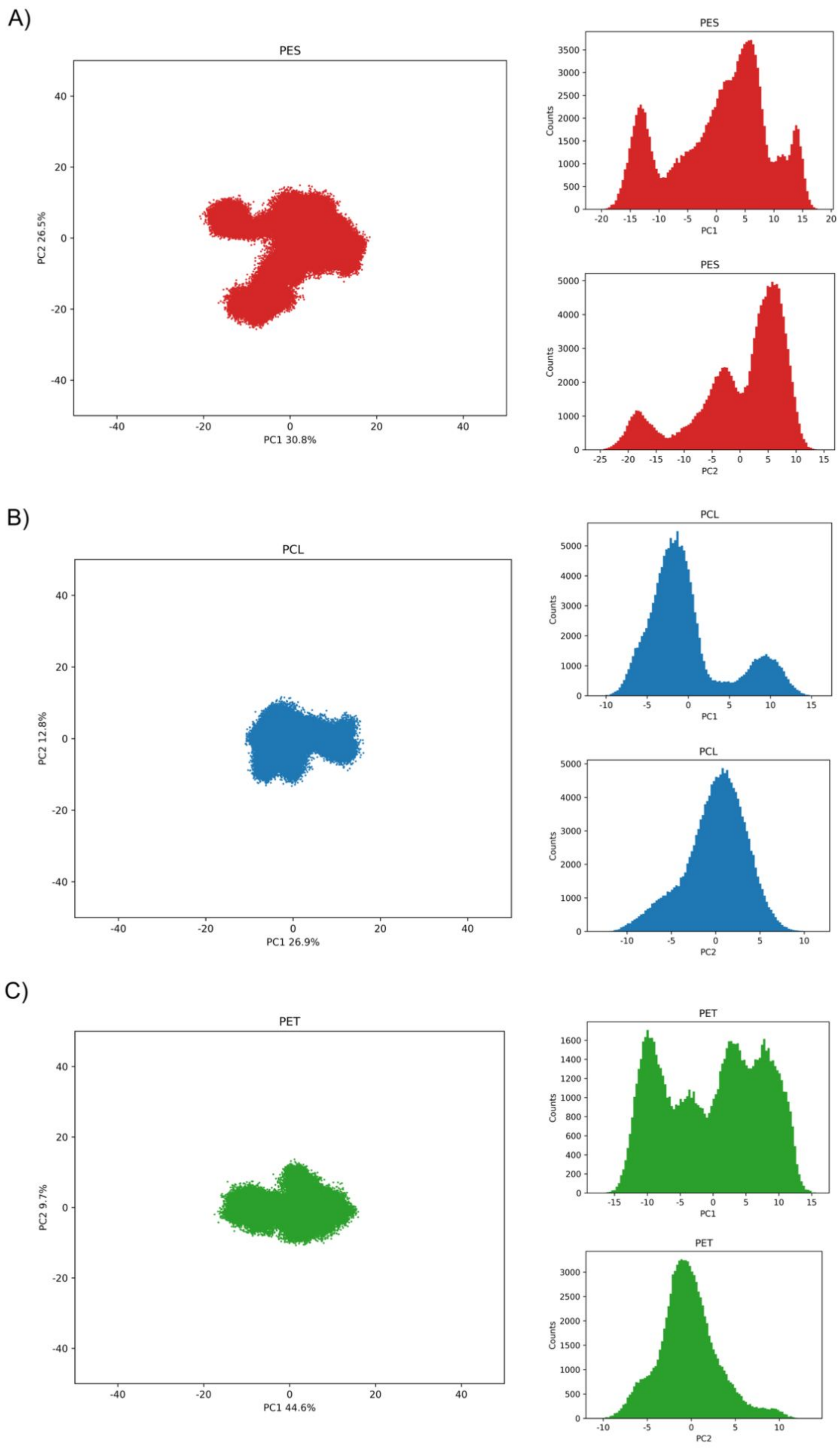

Figure S8. PCA projections (PC1 vs PC2) and 1D histograms of the first two principal components for complexes *Mr*Cut3 with A)PES, B)PCL, and C)PET.

Supplementary References

(Ref. S1) Naïm, M.; Bhat, S.; Rankin, K. N.; Dennis, S.; Chowdhury, S. F.; Siddiqi, I.; Drabik, P.; Sulea, T.; Bayly, C. I.; Jakalian, A.; Purisima, E. O. Solvated Interaction Energy (SIE) for Scoring Protein-Ligand Binding Affinities. 1. Exploring the Parameter Space. *J. Chem. Inf. Model.* **2007**, *47* (1), 122–133. https://doi.org/10.1021/ci600406v.

(Ref. S2) de Oliveira, M. V. D.; da Costa, K. S.; Silva, J. R. A.; Lameira, J.; Lima, A. H. Role of <scp>UDP-N</Scp> -acetylmuramic Acid in the Regulation of <scp>MurA</Scp> Activity Revealed by Molecular Dynamics Simulations. *Protein Science* **2024**, *33* (4). https://doi.org/10.1002/pro.4969.

(Ref. S3) Silva, J. R. A.; Bishai, W. R.; Govender, T.; Lamichhane, G.; Maguire, G. E. M.; Kruger, H. G.; Lameira, J.; Alves, C. N. Targeting the Cell Wall of Mycobacterium Tuberculosis : A Molecular Modeling Investigation of the Interaction of Imipenem and Meropenem with L , D - Transpeptidase 2. *J. Biomol. Struct. Dyn.* **2016**, *34* (2), 304–317. https://doi.org/10.1080/07391102.2015.1029000.

(Ref. S4) Brandsdal, B. O.; Österberg, F.; Almlöf, M.; Feierberg, I.; Luzhkov, V. B.; Åqvist, J. B. T.-A. in P. C. Free Energy Calculations and Ligand Binding. In *Protein Simulations*; Academic Press, 2003; Vol. 66, pp 123–158. https://doi.org/https://doi.org/10.1016/S0065-3233(03)66004-3.

(Ref. S5) Garcia-Mira, M. M.; Sadqi, M.; Fischer, N.; Sanchez-Ruiz, J. M.; Muñoz, V. Experimental Identification of Downhill Protein Folding. *Science* **2002**, *298* (5601), 2191–2195. https://doi.org/10.1126/science.1077809.

(Ref. S6) Gruebele, M. Downhill Protein Folding: Evolution Meets Physics. *C. R. Biol.* **2005**, *328* (8), 701–712. https://doi.org/10.1016/j.crvi.2005.02.007.

(Ref. S7) Bryngelson, J. D.; Onuchic, J. N.; Socci, N. D.; Wolynes, P. G. Funnels, Pathways, and the Energy Landscape of Protein Folding: A Synthesis. *Proteins* **1995**, *21* (3), 167–195. https://doi.org/10.1002/prot.340210302.

(Ref. S8) Karamzadeh, R.; Karimi-Jafari, M. H.; Sharifi-Zarchi, A.; Chitsaz, H.; Salekdeh, G. H.; Moosavi-Movahedi, A. A. Machine Learning and Network Analysis of Molecular Dynamics Trajectories Reveal Two Chains of Red/Ox-Specific Residue Interactions in Human Protein Disulfide Isomerase. *Sci. Rep.* **2017**, *7* (1), 3666. https://doi.org/10.1038/s41598-017-03966-5.

(Ref. S9) Papaleo, E.; Mereghetti, P.; Fantucci, P.; Grandori, R.; De Gioia, L. Free-Energy Landscape, Principal Component Analysis, and Structural Clustering to Identify Representative Conformations from Molecular Dynamics Simulations: The Myoglobin Case. *J. Mol. Graph. Model.* **2009**, *27* (8), 889–899. https://doi.org/10.1016/j.jmgm.2009.01.006.

(Ref. S10) Costa, C. H. S.; Oliveira, A. R. S.; dos Santos, A. M.; da Costa, K. S.; Lima, A. H. L. e.; Alves, C. N.; Lameira, J. Computational Study of Conformational Changes in Human 3-Hydroxy-3-Methylglutaryl Coenzyme Reductase Induced by Substrate Binding. *J. Biomol. Struct. Dyn.* **2019**, *37* (16), 4374–4383. https://doi.org/10.1080/07391102.2018.1549508.

(Ref. S11) Costa, C. H. S. da; Bichara, T. W.; Gomes, G. C.; dos Santos, A. M.; da Costa, K. S.; Lima, A. H. L. e.; Alves, C. N.; Lameira, J. Unraveling the Conformational Dynamics of Glycerol 3-Phosphate Dehydrogenase, a Nicotinamide Adenine Dinucleotide-Dependent Enzyme of Leishmania Mexicana. *J. Biomol. Struct. Dyn.* **2020**, 1–12. https://doi.org/10.1080/07391102.2020.1742206.

(Ref. S12) Grosso, M.; Kalstein, A.; Parisi, G.; Roitberg, A. E.; Fernandez-Alberti, S. On the Analysis and Comparison of Conformer-Specific Essential Dynamics upon Ligand Binding to a Protein. *J. Chem. Phys.* **2015**, *142* (24), 245101. https://doi.org/10.1063/1.4922925.

(Ref. S13) Costa, C. H. S.; Santos, A. M.; Alves, C. N.; Martí, S.; Moliner, V.; Santana, K.; Lameira, J. Assessment of the PETase Conformational Changes Induced by Poly(Ethylene Terephthalate) Binding. *Proteins: Structure, Function, and Bioinformatics* **2021**, *89* (10), 1340–1352. https://doi.org/10.1002/prot.26155.

(Ref. S14) Le Guilloux, V.; Schmidtke, P.; Tuffery, P. Fpocket: An Open Source Platform for Ligand Pocket Detection. *BMC Bioinformatics* **2009**, *10* (1), 168. https://doi.org/10.1186/1471-2105-10-168.

(Ref. S15) Sumbalova, L.; Stourac, J.; Martinek, T.; Bednar, D.; Damborsky, J. HotSpot Wizard 3.0: Web Server for Automated Design of Mutations and Smart Libraries Based on Sequence Input Information. *Nucleic Acids Res.* **2018**, *46* (W1), W356–W362. https://doi.org/10.1093/nar/gky417.
